# An integrated assessment of the ADME properties of the CDK4/6 Inhibitor ribociclib utilizing preclinical in vitro, in vivo, and human ADME data

**DOI:** 10.1002/prp2.599

**Published:** 2020-06-10

**Authors:** Alexander D. James, Hilmar Schiller, Cyrille Marvalin, Yi Jin, Hubert Borell, Ad F. Roffel, Ulrike Glaenzel, Yan Ji, Gian Camenisch

**Affiliations:** ^1^ PK Sciences (ADME) Novartis Institutes for Biomedical Research Basel Switzerland; ^2^ PK Sciences (Oncology TA) Novartis Institutes for Biomedical Research East Hanover USA; ^3^ PRA Health Sciences, Scientific and Medical Affairs Groningen the Netherlands

**Keywords:** ADME, human, Kisquali, preclinical, Ribociclib

## Abstract

Ribociclib (LEE011, Kisqali ®) is a highly selective small molecule inhibitor of cyclin‐dependent kinases 4 and 6 (CDK4/6), which has been approved for the treatment of advanced or metastatic breast cancer. A human ADME study was conducted in healthy male volunteers following a single oral dose of 600 mg [^14^C]‐ribociclib. Mass balance, blood and plasma radioactivity, and plasma ribociclib concentrations were measured. Metabolite profiling and identification was conducted in plasma, urine, and feces. An assessment integrating the human ADME results with relevant in vitro and in vivo non‐clinical data was conducted to provide an estimate of the relative contributions of various clearance pathways of the compound. Ribociclib is moderately to highly absorbed across species (approx. 59% in human), and is extensively metabolized in vivo, predominantly by oxidative pathways mediated by CYP3A4 (ultimately forming N‐demethylated metabolite M4) and, to a lesser extent, by FMO3 (N‐hydroxylated metabolite M13). It is extensively distributed in rats, based on QWBA data, and is eliminated rapidly from most tissues with the exception of melanin‐containing structures. Ribociclib passed the placental barrier in rats and rabbits and into milk of lactating rats. In human, 69.1% and 22.6% of the radiolabeled dose were excreted in feces and urine, respectively, with 17.3% and 6.75% of the ^14^C dose attributable to ribociclib, respectively. The remainder was attributed to numerous metabolites. Taking into account all available data, ribociclib is estimated to be eliminated by hepatic metabolism (approx. 84% of total), renal excretion (7%), intestinal excretion (8%), and biliary elimination (1%).

**Abbreviations**
ADME: Absorption, Distribution, Metabolism, and Excretion
AMS: Accelerator Mass Spectrometry
AUC: Area under the concentration time curve
CRO: Contract Research Organization
CYP: Cytochrome P450
DDI: Drug Drug interaction
FMO: Flavin‐containing monooxygenase
HPLC: High‐performance liquid chromatography
LSC: Liquid scintillation counting
MS/MS: Tandem mass spectrometry
PK: Pharmacokinetics
QWBA: Quantitative whole body autoradiography

## 1 INTRODUCTION

Ribociclib (LEE011, Kisqali ®) is an orally available, highly selective small‐molecule inhibitor of cyclin‐dependent kinases 4 and 6 (CDK4/6). The compound has been approved by a number of health authorities, including the United States Food and Drug Administration (US FDA) and the European Medicines Agency, for the treatment of women with hormone receptor (HR)‐positive, human epidermal growth factor receptor 2 (HER2)‐negative advanced or metastatic breast cancer in combination with an aromatase inhibitor (AI) or fulvestrant.^1^, ^2^, ^3^ Additional marketing authorizations are under review by health authorities worldwide.

During drug development, a human ADME study, in which six male healthy volunteers were administered 600 mg of [^14^C]‐labeled ribociclib was conducted. The human ADME study is essential to identify the major circulating drug‐related components in order to assess any potential quantitative and/or qualitative differences in metabolism between humans and the animal species used in non‐clinical safety assessment. The importance of the study is discussed extensively in various regulatory guidances^4^, ^5^ as well as in the literature.^6^, ^7^, ^8^ Furthermore, the study provides key data that can be used to estimate the main elimination pathways. Correlation of these data with in vitro phenotyping experiments allows a quantitative assessment of the enzymes responsible for the majority of metabolic elimination which, together with the identification of the major circulating metabolites, has important consequences for the understanding of possible drug drug interaction (DDI) liabilities.^9^, ^10^

The objectives of the human ADME study of ribociclib were (a) to determine the rates and routes of excretion of [^14^C]‐ribociclib‐related radioactivity (mass balance) following a single oral dose of 600 mg [^14^C]‐ribociclib to six healthy male subjects, (b) to determine the pharmacokinetics (PK) of total radioactivity in blood and plasma, (c) to characterize the plasma PK of ribociclib and N‐desmethyl metabolite M4 (LEQ803), (d) to characterize the urine concentrations of ribociclib and LEQ803, (e) to identify and quantify ribociclib and its metabolites in excreta in order to elucidate key biotransformation pathways and clearance mechanisms, (f) to characterize the plasma PK of ribociclib and metabolites based on radiometry data, and (g) to assess the safety of a single 600 mg oral dose of [^14^C]‐ribociclib administered to healthy male subjects.

The purpose of this article is to describe the design and results of the human ADME study for ribociclib. In addition, the results of relevant in vitro studies and in vivo radiolabeled animal ADME studies are briefly described. Finally, an integrated assessment of all relevant data was performed in order to estimate the relative contributions of the various clearance pathways of ribociclib in humans.

## 2 MATERIALS AND METHODS

### 2.1 Non‐clinical ADME studies

#### 2.1.1 Pharmacokinetic studies

ADME studies (including QWBA in pigmented and non‐pigmented rats) using radiolabeled ribociclib were conducted in rat and dog. Relevant institutional and national guides for the care and use of laboratory animals were followed. Exposure of organs to total radioactivity was measured by QWBA of tissue sections.^11^ Placental transfer in rat and rabbit was assessed by comparison of ribociclib concentrations in maternal and fetal plasma. Details of the dosing routes, formulations used, and the sampling schedules are provided in Table S1. The synthetic route of [^3^H] ‐ and [^14^C]‐ribociclib, which were used in selected ADME studies, is described in Table S4.

In these studies, PK of ribociclib in plasma, and total radioactivity analysis in blood, plasma, urine, and feces were measured by a combination of validated bioanalytical assays and LSC measurements. Metabolite profiling was conducted on plasma, urine, bile, and feces samples in the rat and dog ADME studies, and further metabolite profiling on plasma and milk was conducted in a dedicated rat milk excretion study.

Plasma samples obtained from these studies were extracted with equal volumes of acetonitrile. Additional extractions of the protein pellet with acetonitrile and/or acetone were conducted as needed to maximize radioactivity recovery. Feces samples were initially extracted with two to four volumes of acetonitrile. Additional extractions of the pellet following centrifugation and removal of supernatant were conducted with acetonitrile and/or acetone as needed to maximize radioactivity recovery. Urine and bile samples were analyzed directly following centrifugation. Radioactivity recoveries following the centrifugation step were measured by LSC. Milk samples were extracted with two volumes of acetonitrile, following centrifugation and removal of supernatant, the pellets were re‐suspended in 100 µL of water and re‐extracted with 200 µL acetonitrile.

QWBA studies were conducted with the tritium label in Hanover Wistar and partially pigmented Long‐Evans rats. A further QWBA study was conducted with the carbon‐14 label in male Long Evans rats in order to support the dosimetry calculation for the human ADME study. Briefly, 40‐µm thick lengthwise dehydrated whole body sections were exposed for 1 day to Fuji BAS III imaging plates (Fuji Photo Film Co., Ltd., J‐Tokyo) in a lead‐shielded box and room temperature, and scanned in a Fuji BAS 5000 phosphor imager at a 50‐µm scanning step. Concentrations of total radiolabeled components in the tissues were determined by comparative densitometry and digital analysis of the autoradiogram; blood samples of known radioactivity concentrations processed under the same conditions as the samples to analyze were used as calibrators.

Metabolite profiling in the ADME studies was conducted using liquid chromatography‐mass spectrometry. Radioactivity profiles were generated by diversion of the majority of the post‐column flow into 96‐well yttrium silicate scintillation‐coated plates (Deepwell Lumaplates; Perkin Elmer Life and Analytical Sciences Inc), by means of Gilson FC204 fraction collectors (Gilson). Metabolites were identified by mass spectrometry (Waters QTOF operating under MassLynx 4.1) and, where feasible, by comparison with authentic reference standards.

Placental transfer of ribociclib was assessed in rat and rabbit embryofetal development toxicology studies. A single plasma sample on the last day of dosing in both studies, at 3 hours post dose, was taken from the fetuses and ribociclib was measured using a validated bioanalytical assay. Resulting concentrations were compared with maternal plasma concentrations. In vitro blood distribution of [^3^H]‐ribociclib was investigated at the nominal blood concentrations of 100, 1000, and 10 000 ng/mL (rat and dog) and at 10, 100, 1000, and 10 000 ng/mL (human). Heparinized blood was spiked with [^3^H]‐ribociclib and incubated for 1 h at 37°C with constant agitation. After the incubation, blood cells and plasma were separated by centrifugation (1500 g, 10 min, 37°C). Total radioactivity was measured by LSC on triplicate aliquots, taken before (blood) and after (plasma) centrifugation. The hematocrit of the whole blood was determined in triplicate after centrifugation in micro hematocrit capillaries (13 000 g, 5 min). Calculation of f_p_ and C_bc_/C_p_ is described in Table S5.

In vitro plasma protein binding of [^3^H]‐ribociclib was investigated at nominal plasma concentrations of 100 and 1000 ng/mL (rat and dog) and at 10, 100, 1000, and 10 000 ng/mL (human). Stock solutions were spiked into plasma to achieve the intended concentrations. After incubation for 1 hour at 37°C under constant gentle agitation, the spiked plasma samples (n = 3) were centrifuged (2000 g, 10 min, 37°C) in pre‐warmed Centrifree devices. Total radioactivity was determined in the ultrafiltrate (C_u_, concentration of unbound compound) and in the sample introduced into the reservoir before ultrafiltration (C_p_). The unbound (f_u_) and the bound (f_b_) fraction in plasma were calculated as follows: f_u_ (%) = C_u_/C_p_ × 100; f_b_ (%) = 100‐F_u_ (%).

### 2.2 Human enzyme phenotyping studies

In vitro incubations were carried out in 100‐mM potassium phosphate buffer (pH 7.4) and 5‐mM MgCl_2_ with a total volume of 200 µL. Substrate and either human liver microsomes (HLM, BD Biosciences, mixed gender pool of n = 50) or recombinant human enzymes (BD Biosciences) were added and pre‐incubated for 3 minutes at 37°C. The reaction was started by addition of a fresh solution of NADPH (1 mmol/L final concentration). The samples were incubated at 37°C with agitation of 500 rpm. For incubations with CYP2A6, CYP2C9, CYP2C18, and CYP4A11, the phosphate buffer was replaced by TRIS buffer (100 mmol/L, pH 7.4) and for flavin‐containing monooxygenases (FMOs), a glycine buffer (50 mM, pH 9.5) was used.

The enzymatic reaction was stopped and the protein was precipitated by addition of an equal volume of methanol. After 30 min at −80°C, the samples were centrifuged at 30'000 x g for 15 min. The supernatant was withdrawn. Aliquots were analyzed by LSC and the supernatant was diluted with water to obtain a final solution containing less than 20% of the organic solvent. For samples of low substrate concentration, supernatants were evaporated to dryness under nitrogen, then re‐suspended in water containing less than 20% of methanol. Samples were analyzed by HPLC combined with radiodetection.

FMO inactivation in HLM: The FMO in HLM was inactivated by heating up the microsomal fraction at 50°C for 1 minute as described below: tubes with the appropriate volume of phosphate buffer and 2 µL 1M MgCl_2_ were pre‐warmed in a water bath at 50°C for at least 5 minutes. 8 µL HLM (20 mg/mL stock solution) were added to each tube (n = 2). All tubes were mixed quickly and incubated for exactly 1 minute at 50°C and immediately cooled down to 0°C in an iced water bath.

Enzyme kinetic parameters Km and Vmax in HLM were determined after establishing linear reaction conditions by incubating ribociclib in pooled HLM at 22 nominal substrate concentrations ranging from 0.125 µmol/L to 300 µmol/L and incubation times of 8 min or 15 min. Km and Vmax parameters were calculated by using SigmaPlot Version 8.0, Enzyme Kinetics module Version 1.1 software (SPSS Science Inc, Chicago, IL, USA). The intrinsic clearance was calculated by the equation: CLint = Vmax/Km.

### 2.3 Human ADME study

#### 2.3.1 Study Drug

The radiolabeled drug [^14^C]‐ribociclib succinate salt was synthesized by the Isotope Laboratory of Novartis, Basel, Switzerland. The synthetic route is described in Table S4. The final drug product was analyzed by the Isotope Laboratory and Pharmaceutical and Analytical Development department of Novartis and was released for human use according to predefined specifications. The chemical and radiochemical purity of the drug was 99.7%, with individual impurities accounting for ≤ 0.15%. The nominal specific radioactivity of [^14^C]‐ribociclib was 0.62 kBq/mg, referring to free base. The study drug was provided as capsules of 200 mg ^14^C‐ribociclib. Three capsules were packed per bottle, which provided the dose of 600 mg.

#### 2.3.2 Study Volunteers

This single‐center, open‐label, single oral dose study enrolled six healthy, non‐smoking, male Caucasian volunteers who were determined as being in good health according to their medical history, physical examination, vital signs, electrocardiogram, laboratory tests, and urinalysis. Healthy male volunteers were selected as the foundation of the extensive human ADME data should be based on a small cohort (6) of young healthy male volunteers with subsequent extension/bridging to actual patients as needed for the investigation of variables such as age, gender, ethnicity, and health on the metabolic profile. Subjects with relevant radiation exposure of > 0.2 mSv in the 12 months prior to the initiation of the study were excluded. The subjects were exposed to a radiation dose of 1.77 mSv maximally, which was calculated according to the guidelines of the International Commission on Radiological Protection. The clinical study was performed at PRA Health Sciences, Zuidlaren, the Netherlands, in accordance with Good Clinical Practice guidelines and the 1964 Declaration of Helsinki and subsequent revisions. The study protocol and dosimetry calculations were reviewed by the Independent Ethics Committee for the center, and written informed consent was obtained from all subjects before entering the study.

#### 2.3.3 Safety Assessments

Safety analysis included monitoring and recording of all adverse events, laboratory tests (ophthalmologic exam, hematology, blood chemistry, and urinalysis), vital signs, electrocardiogram, and physical examination.

#### 2.3.4 Dose Administration and Pharmacokinetic Sampling

After an overnight fasting of approximately 10 hours, each subject received a single oral dose of [^14^C]‐ribociclib 600 mg in three capsules of 200 mg each, which were taken consecutively with 1 glass of noncarbonated water. Subjects continued to fast for 4 hours after drug administration. After dosing, blood (plasma), urine, and feces samples were collected for 21 days at 0, 0.25, 0.5, 1, 1.5, 2, 3, 6, 12, 24, 36, 48, 72, 96, 120, 144, 168, 192, 216, 240, 264, 312, 360, 408, 456, and 504 h post dose. Urine was collected from 0‐6, 6‐12, and 12‐24 h post dose, then over 24‐hour intervals until 504 hours post dose. Feces was collected over 24‐h intervals until 504 hours post dose, during which time the volunteers were confined to the clinic. Vomitus was also scheduled to be collected for 12 hours post dosing. However, no volunteers vomited during the study.

#### 2.3.5 PK of ribociclib and Metabolite LEQ803 in Plasma and Urine

Ribociclib and LEQ803 were measured in plasma and urine using a validated bioanalytical assay. Plasma aliquots at each time point were subjected to protein precipitation with three volumes of acetonitrile containing 0.1% (v:v) formic acid, followed by dilution and analysis by liquid chromatography‐tandem mass spectrometry in selected reaction monitor‐positive ion mode using heated electrospray ionization as the ionization technique. For urine aliquots, six volumes of acetonitrile containing 0.1% (v:v) formic acid were added, followed by dilution and analysis in the same way as for plasma. Components were separated using a YMC‐Triart C18 (2.0 x 30 mm, 1.9 µm) column (YMC Co. Ltd.). Mobile phase A was held at 95% for 0.5 min, then reduced to 80% at 0.8 min, 60% at 2 min, and 5% at 2.5 min, where it was held until 4 min. Finally, it was increased to 95% at 4.1 min. Mobile phase A was 0.1% formic acid in H_2_O while mobile phase B was 0.1% formic acid in ACN:Isopropanol 8:2 v:v. The validated range for ribociclib and LEQ803 in plasma and urine was 1.0 (LLOQ) and 1000 ng/mL (ULOQ), using 50 µL of sample per analysis.

#### 2.3.6 Total radioactivity measurements

Total radioactivity in blood and plasma was analyzed by accelerator mass spectrometry (AMS) on a National 5.3.2. Electrostatics Corporation 1.5SDH Compact AMS System (Middleton) in the bioanalytical laboratory of Accium BioSciences, Seattle, WA, USA. A known aliquot of each blood and plasma specimen was transferred to a prebaked quartz tube. Sample volumes were selected to achieve approximately 1–2 mg total carbon. An AMS batch consisted of unknown specimens and chemical blank(s) to monitor for any in‐process contamination. The samples were then dried using vacuum centrifugation and submitted to graphitization. Graphitization consisted of combustion of samples followed by reduction to graphite according to published methods.^12^ Briefly, approximately 200‐mg copper oxide was added to each combustion tube containing the dried sample residue. The combustion tubes were flame‐sealed under vacuum and combusted at approximately 900°C to form carbon dioxide (CO_2_). Combusted samples were then attached to a disposable transfer system connected to a septa‐sealed vial. This vial contained a minimum of 100 mg of zinc powder, several 3‐mm glass beads, and a smaller vial containing 2‐ to 6‐mg iron powder. The transfer system was evacuated with a vacuum pump and the tip of the combustion tube was broken to allow release of gases into the septa‐sealed vial, which was submerged slightly in liquid nitrogen. Gases, such as CO_2_ and H_2_O, condensed at the bottom of the vial while noncondensing gases were purged using the vacuum pump. To avoid cross‐contamination, all parts that came into contact with the sample were disposed of and replaced with each use. The septa‐sealed vials were then placed in a heat block maintained between 515°C and 525°C for approximately 5 hours. During this stage, carbon from CO_2_ reduced to solid graphite, adhering to the surface of the iron powder. The resulting iron‐graphite mixture was pressed into individual cathodes and submitted for AMS measurement. Pressed, individual cathodes were loaded on to the AMS sample wheel for measurement. A typical AMS measurement batch contained unknown samples, certified standards to normalize all measurements, machine blanks ([^14^C]‐free graphite of natural origin) to assess the sensitivity of the spectrometer, and chemical blanks (blanks prepared with a [^14^C]‐free substance) to characterize the extraneous carbon introduced during graphite batch preparation.

Radioactivity contents in urine and feces homogenates were determined at the bioanalytical laboratory of PRA International, Zuidlaren, the Netherlands. For urine, duplicate (1000 µL) aliquots were placed into 7‐mL glass vials (Perkin Elmer), after which 5 mL of scintillation cocktail (Ultima Gold™, Perkin Elmer) was added. After vortex mixing for 5 seconds, each sample was placed in a Tri‐Carb™ 3100 TR liquid scintillation analyzer (Perkin Elmer, Waltham, MA, USA) 30 minutes before counting. The total [^14^C]‐radioactivity of the samples was determined by counting until a statistical error (two standard deviations) of 0.5% was obtained with a counting time of 10 or 30 minutes, depending on the level of radioactivity.

For feces homogenates, quadruplicate, accurately weighed (500 mg) aliquots were dried in a stove at 50ºC for 3 hours. After the addition of 100‐µL combustion aid (Perkin Elmer) to the dry homogenates, the samples were combusted in a sample oxidizer model 307 (Perkin Elmer). The absorber agent for CO_2_ was 7‐mL Carbo‐Sorb® E (Perkin Elmer). At the end of the combustion cycle, the absorber was mixed with 13 mL of the scintillant Permafluor® E (Perkin Elmer). The samples were placed in the liquid scintillation analyzer for 60 minutes before counting. The total [^14^C]‐radioactivity of the samples was determined by counting until a statistical error (2 s) of 0.5% was obtained with a counting time of 10 or 30 minutes, depending on the level of radioactivity.

#### 2.3.7 Quantitative metabolite profiling in plasma

A plasma pool across the six healthy volunteers included in the study was prepared at the time points of 1 hour, 3 hours, 24 hours, and 48 hours post dose. For each time point, an equal volume of plasma was taken from each subject and combined. Plasma pool samples were processed by protein precipitation with acetonitrile. The protein pellet that resulted from precipitation with acetonitrile was washed further with additional aliquots of acetonitrile. All acetonitrile washes were combined and evaporated under nitrogen. For the 3‐ and 24‐hour plasma pools, the extraction procedure was slightly modified, including precipitation of the plasma proteins by addition of a 1:1 mixture of acetonitrile:methanol, followed by washing of the protein pellet with the same solvent mixture. All extracts were reconstituted with a solution of 15‐mM ammonium formate and acetonitrile (95:5 v/v). Extraction and reconstitution recoveries were measured in all four plasma samples in order to calculate and overall recovery.

Quantitative metabolite profiling in plasma was accomplished using a Shimadzu Prominence high‐performance liquid chromatography (HPLC) system (Shimadzu, Columbia, MD, USA), coupled with fraction collection and analysis of total radioactivity in individual fractions using AMS (as described above). A second injection of each sample, conducted immediately after the first, was used to provide HPLC fractions for metabolite identification. The fractions identified as containing radioactivity from the AMS analysis were analyzed by HPLC coupled with high‐resolution mass spectrometry to identify the metabolites. The HPLC‐MS/MS methodology used is described below.

#### 2.3.8 Quantitative metabolite profiling in urine and feces

For each subject, urine and feces pools were created by combining identical percentages of the volumes of the different excreta fractions, with the objective of representing> 95% of the radioactivity eliminated via each route. Average pools across the six subjects were also created by combining equal percentages (of total urine and feces excreted over the time interval) of the individual subject pools. Urine pools were analyzed directly for metabolite profiling (column recovery of a representative sample was measured), whereas feces pools were subjected to extraction. Specifically, to each aliquot of feces homogenate, two volumes of acetonitrile were added. After centrifugation, the pellet was washed further with an additional two volumes of acetonitrile. Additional pellet wash steps were done with methanol, dimethyl sulfoxide, acetone, and dichloromethane. All washes were combined, evaporated under nitrogen, and reconstituted with a solution of 15‐mM ammonium formate (pH 3.5). This feces extract reconstitute was analyzed for metabolite profiling. Combined extraction and reconstitution (overall recovery) and column recovery were measured.

Metabolite profiling in urine and feces was accomplished using an Agilent model 1200 HPLC (Agilent Technologies, Basel, Switzerland) coupled with a Waters Synapt‐G2‐S HDMS (Waters, Milford, MA, USA) and a Gilson fraction collector GX‐271 (Gilson Inc, Middleton, WI, USA). The HPLC column used was a Phenomenex Kinetex, C18, 250 × 4.6 mm, equipped with a pre‐column of the same type (2.1 × 4.6 mm), which was placed in a column oven at 40°C. Separation of components was achieved using 15‐mM ammonium formate solution in water adjusted at pH 3.5 with formic acid as mobile phase A and acetonitrile:methanol (9:1, v/v) as mobile phase B. Flow rate was 1.25 mL/min. The HPLC gradient was as follows: initial conditions were 5% mobile phase B for 1 minute, then increased to 20% at 10 minutes post injection where it was held (isocratic) up to 20 minutes. From there, mobile phase B was increased to 35% at 30 minutes, and to 100% to 35 minutes where it was held (isocratic) up to 45 minutes. The post column flow was directed to the fraction collector operated in a time‐slice mode and containing 96‐well Lumaplates (Perkin Elmer). The plates were dried at room temperature and the radioactivity was measured using a microplate scintillation counter model TopCount NXT (Perkin Elmer) instrument (up to 3 × 100‐minute counting time). Chromatograms were evaluated using Microsoft Excel 2010. Metabolites were identified during the same injections using data generated by the high‐resolution mass spectrometer. The molecular ions and key fragments of each drug‐related component were determined. Key fragments for each metabolite were derived from their product ion mass spectra and were used to determine the metabolite structures. Where possible, structural assignments were supported by exact mass measurement, comparison with synthetic standards and/or Hydrogen/Deuterium (H/D) exchange LC‐MS(/MS).

## 3 RESULTS

### 3.1 Non‐clinical ADME:

#### 3.1.1 Absorption

Pharmacokinetic parameters in rat and dog are listed in Table S2. Plasma clearance was high in rats (3.1 L/h/kg in males and 7.8 L/h/kg in females) and dogs (1.9 L/h/kg), and volume of distribution was large (7.9 L/kg in rat and 27.9 L/kg in dog). Elimination half‐life was moderate in rats (1.9‐3.2 h) and long in dog (18.1 h). Bioavailability was 37%‐55% in rat and 64%‐87% in dog. Ribociclib showed a moderate first‐pass effect in rats, with 66% absorption and 37% bioavailability.

#### 3.1.2 Distribution

*R*ibociclib blood‐to‐plasma concentration ratios (Cb/Cp) were independent of concentration in preclinical species and humans up to 10,000 ng/mL. Cb/Cp was species dependent, with rat (0.90 ± 0.01) < human (1.01 ± 0.08) < dog (1.30 ± 0.00). Plasma protein binding was moderate in all tested species, with no concentration dependency observed in the range between 100 and 1000 ng/mL (rat, dog) or the range between 10 and 10 000 ng/mL (human). The unbound fraction in plasma showed up to 1.7‐fold difference between species, with rat (0.20 ± 0.01) < human (0.30 ± 0.02) ≈ dog (0.34 ± 0.01).

Tissue distribution of ribociclib was studied in rats. Based on quantitative whole‐body autoradiography, total radioactivity showed marked distribution into the extravascular compartment, except for brain, after intravenous or oral dose administration, followed by rapid elimination from most tissues. In pigmented animals, specific distribution of radioactivity to melanin‐containing structures (eg, eye choroid, meninges) was observed. The time of last measurable concentration was short (≤ 48 hours) for 52 of 58 tissues investigated, but longer for the lymph nodes, preputial gland, testis (168 hours), eyes, and meninges (measurable up to 840 hours [ie, the last observation time point]). Ribociclib was found to pass the placental barrier in rats and rabbits. Fetal plasma concentrations were 6%‐29% of maternal plasma concentrations in rats and 6% in rabbits, based on samples taken at 3 hours post dose on Days 17 and 20 postcoitum, respectively. Ribociclib also passed into the milk of lactating rats, where exposure to ribociclib (AUCinf) was found to be 3.6‐fold higher than plasma exposure.

#### 3.1.3 Metabolism

Metabolism of ribociclib was investigated in rat and dog. Predominant metabolic pathways in the rat were direct conjugation to the sulfate metabolite M8 and N‐dealkylation to LEQ803 with subsequent Phase II reactions. In dog, metabolism was dominated by oxidative pathways like dealkylation, C‐ and N‐oxygenation, oxidations, and combinations of these reactions. In both species, the major plasma component was unchanged ribociclib. Metabolite LEQ803 was found in the circulation of both species and represented between 3% (dog) and 38% (rat) of plasma exposure to ribociclib. A higher ratio of LEQ803 to ribociclib AUC after p.o. compared to iv dosing in rat and dog indicated a first pass effect in generation of this metabolite. The higher clearance in female rats compared to males was attributed to a more pronounced metabolism to the sulfate metabolite M8 in females.^13^ The formation of M8 was a minor metabolic pathway in dog. Representative metabolite profiles from rat and dog ADME studies are provided in Figure S3 and Figure S4.

#### 3.1.4 Excretion

In rat and dog, the predominant route of elimination was fecal. Following oral dosing of radiolabeled ribociclib, 84.0 and 68.8% of the radioactivity was found in feces and 5.9 and 18.5% in urine in rat and dog, respectively. Excretion was fast in rat, but slow in dog and human in line with the plasma elimination half‐life.

Ribociclib was eliminated in both species mainly by metabolism with limited contribution of renal clearance. After p.o. administration, unchanged ribociclib in urine represented 2.7 and 13.4% of the dose in rat and dog, respectively. In feces, ribociclib accounted for 22.4 and 10.7% of the oral dose in rat and dog, respectively. In rat bile following i.v. administration, only 1% of the dose was recovered as unchanged ribociclib (61.4% of dose was eliminated in bile), indicating that biliary excretion of metabolites is the major excretion route in rat. Evidence for intestinal secretion of ribociclib was observed as ribociclib in the feces of bile duct‐cannulated rats amounted to 8% of the dose after i.v. administration. Recovery of radioactivity in mass balance studies was good (approximately 90%) in both species.

### 3.2 Enzyme phenotyping

In vitro oxidative metabolism of [^3^H] ribociclib was investigated in human liver microsomes (HLM) and recombinant human CYP and FMO enzymes in the presence of NADPH. In HLM, [^3^H] ribociclib was mainly metabolized by hydroxylation (M15), hydroxylamine formation (CCI284), and N‐demethylation (M4, LEQ803) as shown in Figure S1.

Enzyme kinetic parameters Km and Vmax were determined in pooled HLM. The kinetic data were best fitted using the Michaelis‐Menten model (Figure S2) with a Km of 29.1 ± 1.8 µmol/L and a Vmax of 766 ± 13 pmol/min/mg. The derived intrinsic clearance (Vmax/Km) of the total oxidative hepatic metabolism of ribociclib was 26.4 µL/mg/min.

As FMOs are more thermally labile than CYPs in the absence of NADPH,^14^ heat treatment of HLM was used to estimate the contribution of FMO. HLM was heated to 50°C for 1 min to inactivate the FMO activity. Figure S1 shows the effect of heat treatment. Formation of the hydroxylamine CCI284 was almost completely abolished after 1 min heating at 50°C, whereas no significant changes were seen for other metabolites. This result indicated a predominant role of hepatic FMO in the N‐oxidation of ribociclib. Enzyme kinetics in heat‐treated HLM (0.4 mg/mL) was performed by incubation with 12 nominal concentrations of ribociclib between 0.5 µmol/L and 200 µmol/L for 15 min. Enzyme kinetic data were best fitted using the substrate inhibition kinetic model resulting in a total metabolism Km of 12.1 ± 4.8 µmol/L and a Vmax of 273 ± 46 pmol/min/mg. The Ki value was 328 µmol/L. The derived intrinsic clearance (Vmax/Km) was 22.5 µL/mg/min. Comparing the intrinsic clearance in heated HLM to that of non‐heated control HLM, it was estimated that FMO metabolism constitutes about 15% of the intrinsic clearance in HLM.

To identify the major metabolizing CYPs or FMOs in human liver microsomes, correlation analyses were conducted in liver microsomes from 16 individual donors. Metabolic rates of [^3^H] ribociclib (1.5 µmol/L and 20 µmol/L) in the 16 single donor HLM were correlated with CYP and FMO marker enzyme activities provided by the vendor (Table S3). [^3^H] ribociclib total metabolism correlated best with CYP3A4/5 activities with correlation coefficients (R) of 0.975 and 0.893 for testosterone 6β‐hydroxylase and midazolam 1′‐hydroxylase activities, respectively (Table S3). Formation of the hydroxylamine metabolite CCI284 correlated best with the FMO activity (R = 0.764, Table S3), suggesting a predominant involvement of FMO for the N‐oxide formation in HLM. Formation of M15, M4 (LEQ803), and minor peaks correlated strongly with CYP3A4/5 activity (Table S3), indicating that these metabolites are products of CYP3A4/5.

**Figure 1:**
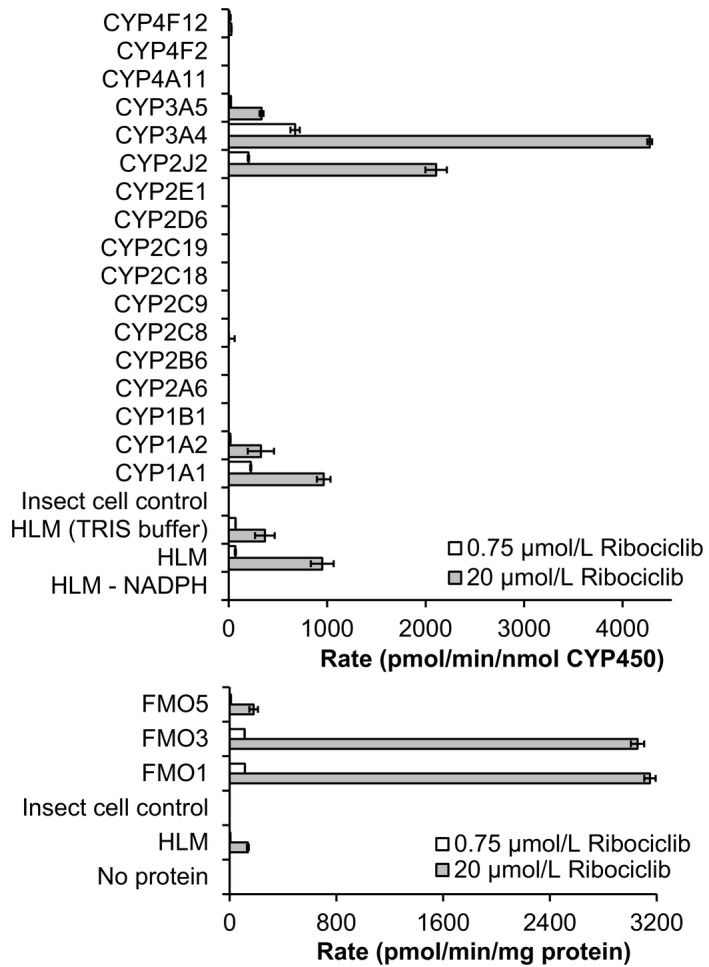
Biotransformation of [^3^H] ribociclib by recombinant human CYP450s and FMOs. The biotransformation of [^3^H] ribociclib by recombinant human enzymes (30 pmol CYP/mL upper figure; and 0.4 mg/mL FMO, low figure) and in HLM (116 pmol CYP/mL) was investigated with an incubation time of 15 min and initial substrate concentration of 0.75 and 20 µmol/L. Formation of the sum of all metabolites was determined by HPLC analysis combined with radiodetection

Involvement of specific enzymes in the biotransformation of 0.75 µmol/L and 20 µmol/L [^3^H] ribociclib was assessed with a panel of 17 recombinant human CYPs and three FMOs ribociclib. At both concentrations (Figure 1), CYP3A4, CYP2J2, CYP1A1, FMO1, and FM03 showed high turnover under the experimental conditions used. Low metabolic activities were also observed in incubations with CYP1A2, CYP3A5, FMO3, and FMO5, while trace metabolism was detected with CYP2C8 and CYP4F12. No metabolic activities were detected with CYP1B1, CYP2A6, CYP2B6, CYP2C8, CYP2C9, CYP2C18, CYP2C19, CYP2D6, CYP2E1, CYP4A11, and CYP4F2.

Enzyme kinetic parameters of ribociclib metabolism by CYP3A4 were determined by incubating [^3^H] ribociclib at different concentrations with the recombinant enzyme. Fitting of the data with the substrate inhibition model provided a Km of 6.68 ± 0.69 µmol/L and a Vmax of 12.3 ± 5.2 pmol/min/pmol. The derived intrinsic clearance (Vmax/Km) for ribociclib by recombinant CYP3A4 is 1.84 µL/pmol CYP/min.

The relative contribution of CYP enzymes to oxidative hepatic metabolism was determined using their Clint values multiplied by the unbound fraction in microsomes and their abundances in human liver microsomes.^15^ Among the hepatic P450 enzymes, CYP3A4 contributed predominantly (97%) to metabolism in human liver microsomes (Table 1) with negligible fraction metabolized by CYP1A2 (1.1%), CYP2J2 (0.8%), and CYP3A5 (1.2%).

**Table 1:** Relative contribution of hepatic P450 isoenzymes to the total CYP‐mediated metabolism in HLM based on recombinant enzyme data

Enzyme	Km	V_max_	fumic	ISEF	Vmax/Km	ISEF*Vmax/Km,u	Abundance^a^	Rel.Abund.	Rel.Clint,u in HLM	fm,CYP
	(µmol/L)	(1/min)			µl/(min. pmol)	µl/(min. pmol)	(pmol P450/mg)		µl/(min.mg)	(%)
CYP1A2	48	1.111	0.86	0,372	0.023	0.010	52	10,2%	0,5	1.1
CYP2A6							20	3,9%		
CYP2B6							17	3,3%		
CYP2C8							24	4,7%		
CYP2C9							73	14,3%		
CYP2C18							1	0,2%		
CYP2C19							14	2,7%		
CYP2D6							8	1,6%		
CYP2E1							61	11,9%		
CYP2J2	11.8	3.55	0.86	1	2.84	0.330	1,2	0,2%	0.4	0.8
CYP3A4	6.68	12.3	0.83	0.157	1.841	0.348	137	26,8%	47.7	97
CYP3A5	23.9	0.732	0.87	0.157	0.031	0.006	103	20,1%	0.6	1.2
total							511	100%	49.2	100

^a^ The abundance of CYPs obtained from ^2004^ and Simcyp.

**Figure 2:**
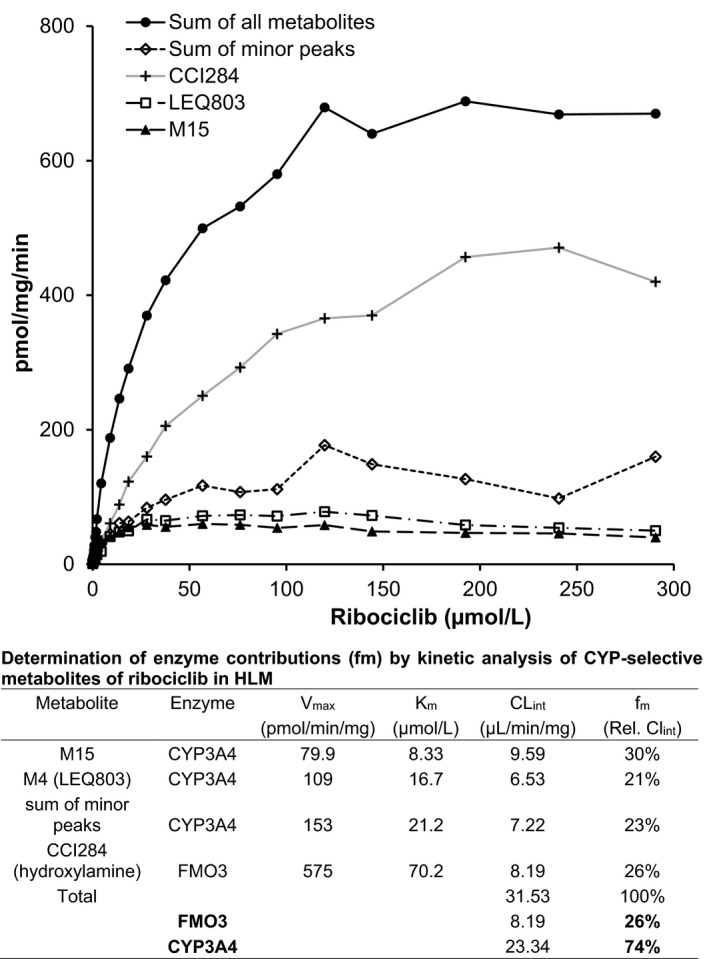
Kinetic analysis of [^3^H] ribociclib metabolite formation in HLM and calculation of enzyme contribution. Concentration‐dependent rate of [^3^H] ribociclib biotransformation to individual metabolites in HLM (0.4 mg protein/mL). Enzyme kinetic parameters were calculated using substrate concentrations of 0.125 µmol/L to 300 µmol/L

Figure 2 shows the metabolic rates of ribociclib metabolite formation in HLM over a wide range of substrate concentrations. The intrinsic clearances (Vmax/Km) of each pathway were determined by enzyme kinetics. The relative CLint (%) of ribociclib metabolism to M15, LEQ803 and minor metabolites represent the fraction metabolized by CYP3A4, while formation of M13 (CCI284) represents the fraction metabolized by FMO. Based on metabolite formation kinetics, 74% of the oxidative metabolism in HLM results from the contribution of CYP3A4, whereas 26% is due to hepatic FMO activity. The effect of CYP‐ and FMO‐selective chemical inhibitors ^16^, ^17^ was determined in human liver microsomes at a ribociclib concentration of 1.5 µmol/L (20 µmol/L for methimazole) (Table 2). The concentration ranges of the inhibitors encompassed reported apparent Ki values (median and ranges) for inhibition of specific CYP. Strong inhibition up to 75% was shown with ketoconazole (CYP3A4 inhibitor). The apparent IC50 value of 0.14 µmol/L is similar to the literature median Ki value for CYP3A4 inhibition by ketoconazole. With azamulin, a more specific inhibitor of CYP3A4,^18^ up to 54% inhibition was obtained with an apparent IC50 of 1.05 µmol/L. Methimazole, a specific inhibitor of FMO, inhibited up to 36% of ribociclib metabolism in this assay. The other chemical inhibitors tested did not show significant effects.

**Table 2:** Inhibition of [^3^H] ribociclib total metabolism by CYP‐ and FMO‐selective inhibitors. The IC50 values of ribociclib metabolism inhibition were compared to reported Ki values of CYP‐specific inhibitors

Inhibitor (CYP)		Concentration range (µmol/L)	Reported K_i_ or IC_50_ values (µmol/L) ^a^ ^b^			IC_50_ (µmol/L)	% maximal inhibition	
Furafylline (1A2)	0.156‐10			2 (0.045‐361)	0.6‐0.73	>10		0
Montelukast (2C8)	0.008‐2			0.014 (0.009‐0.15)		>2		10
Sulfaphenazole (2C9)	0.08‐5			0.51 (0.06‐47)	0.3	>5		6
Ticlopidine (2C19)	0.2‐10			1.7 (0.184‐10)	1.2	>10		3
Quinidine (2D6)	0.008‐2			0.0605 (0.00078‐53)	0.027‐0.4	>2		6
Ketoconazole (3A)	0.008‐1			0.1 (0.001‐32)	0.04‐0.18	0.14		75
Azamulin (3A)	0.04‐5			0.15 (0.12‐0.24)		1.05		54
Methimazole (FMO)	2.5‐160				61^c^	>160		36

^a^ Values from literature search, median (and range)

^b^ Values from Food and Drug Administration (2006)

^c^ Value from Zhou et al (2002)

### 3.3 Human ADME study

#### 3.3.1 Safety evaluation

All subjects received a single oral dose of 600 mg ribociclib. Five subjects experienced at least one adverse event (AE) during the study. The most commonly affected primary system organ class was gastrointestinal disorders (primarily abdominal discomfort [2 subjects, 33.3%]). All AEs were of grade 1 severity. Only one subject had an AE which was suspected to be related to the study drug by the investigator (headache). The AE was resolved on the same day, and the subject did not receive any treatment for this event. No serious adverse events (SAEs) were reported during the study, and no subjects discontinued the study due to an AE.

#### 3.3.2 Mass balance

**Figure 3:**
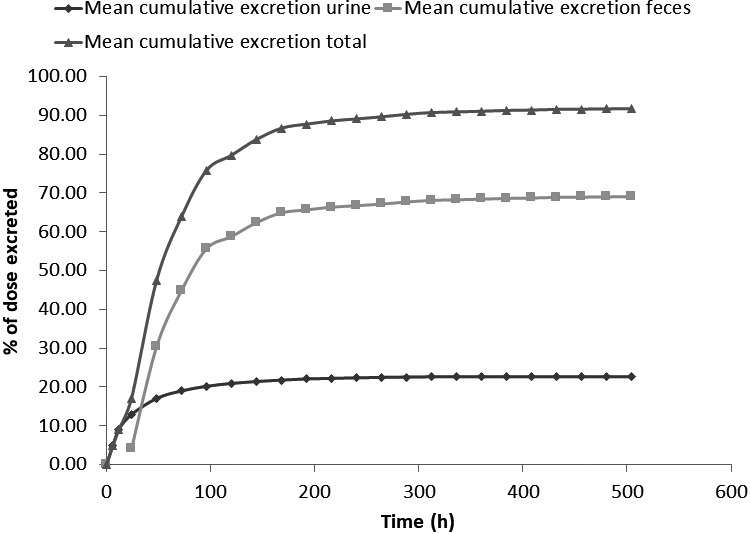
Mean cumulative excretion of radioactivity in urine and feces in the human absorption, distribution, metabolism, and elimination study conducted in healthy male volunteers (N = 6) who received a single oral dose of ^14^C‐ribociclib 600 mg. Source data for Figure 3 are shown in Table S11

Following oral administration of [^14^C]‐ribociclib 600 mg, most of the radioactive dose was excreted in feces (mean 69.1 ± 4.72%) after 504 hours. In urine, mean 22.6 ± 5.39% of the dose was excreted after 504 hours. Mean mass balance in the six volunteers was 91.7 ± 1.01%, which is almost complete. A graphical representation of the cumulative excretion is provided in Figure 3.

#### 3.3.3 Absorption

Oral absorption of ribociclib was estimated to be moderate (ie, approximately 58.8% based on the mean recovery of radioactivity in urine (22.6%) and mean radiolabeled metabolites excreted in feces (36.2%) [assuming all metabolites detected in feces were formed systemically]) Further information can be found in Table S7 and Table S11.

#### 3.3.4 Total Radioactivity (Drug‐Related Material) PK in Blood and Plasma and Ribociclib PK in Plasma

Concentrations of total radioactivity in blood and plasma were measured by AMS, and concentrations of ribociclib in plasma were measured by validated bioanalytical assay, as described above. Results are summarized in Table 3. The median T1/2 of ribociclib in plasma was 49.4 h (arithmetic mean 54.7 h). The T1/2 observed for total radioactivity in plasma was substantially longer (299 h), suggesting possible presence of long‐lived metabolites or a small amount of covalent binding to endogenous plasma components. The mean AUCinf of total radioactivity and ribociclib in plasma was 37 200 and 8700 ng‐eq·h/mL, respectively. The overall contribution of ribociclib to the total radioactivity in plasma, based on mean AUCinf was approximately 23%.

**Table 3:** Summary of pharmacokinetic parameters for total radioactivity in blood and plasma and for ribociclib in plasma in the human absorption, distribution, metabolism, and elimination study conducted in healthy male volunteers (N = 6) who received a single oral dose of [^14^C] ribociclib 600 mg

Parameter		tmax (h) (range)	tlast (h) (range)	t½ (h) (CV %)	Cmax (ng‐eq/mL/ ng/mL)^a^ (CV %)	AUClast (ng‐eq·h/mL/ ng·h/mL)^a^ (CV %)	AUCinf (ng‐eq·h/mL/ ng·h/mL)^a^ (CV %)
Total radioactivity, blood	Mean	‐	‐	240 (20.6)	1280 (31.6)	32,900 (10.9)	36,100 (8.84)
	Median	3 (1‐6)	504	240	1120	32 400	36 100
Total radioactivity, plasma	Mean			293 (24)	1140 (28)	32,500 (16.6)	37,200 (18.3)
	Median	3 (1‐6)	504	299	1060	32 100	37 300
ribociclib, plasma	Mean			54.7 (26)	507 (36.5)	8600 (36.2)	8700 (35.7)
	Median	1.75 (1‐6)	252 (192‐312)	49.4	518	8780	8880
LEQ803, plasma	Mean			91.8 (42.4)	67.6 (36.6)	2160 (21.3)	2320 (22.6)
	Median	2.5 (1‐6)	252 (240‐312)	78.2	59.0	2240	2390

Note

Median values reported for tmax and tlast, including range where existing. Arithmetic mean with coefficient of variation (CV) in %, and median values are reported for t½, Cmax, AUClast, and AUC∞. AUClast, area under the concentration‐time curve from time 0 to time of last measurable concentration; AUC∞, area under the concentration‐time curve from time 0 to infinity; Cmax, maximum concentration; t½, terminal elimination half‐life; tlast, Time of last measured concentration; tmax, time to maximum concentration.

^a^ ng‐eq/mL for radioactivity.

#### 3.3.5 Renal clearance of ribociclib

The median CL/F of ribociclib was 70.2 L/h. The median renal clearance of ribociclib was 5.55 L/h, which was more than 10‐fold lower than the non‐renal clearance (64.7 L/h).

#### 3.3.6 Metabolite Profiles in Plasma

**Figure 4:**
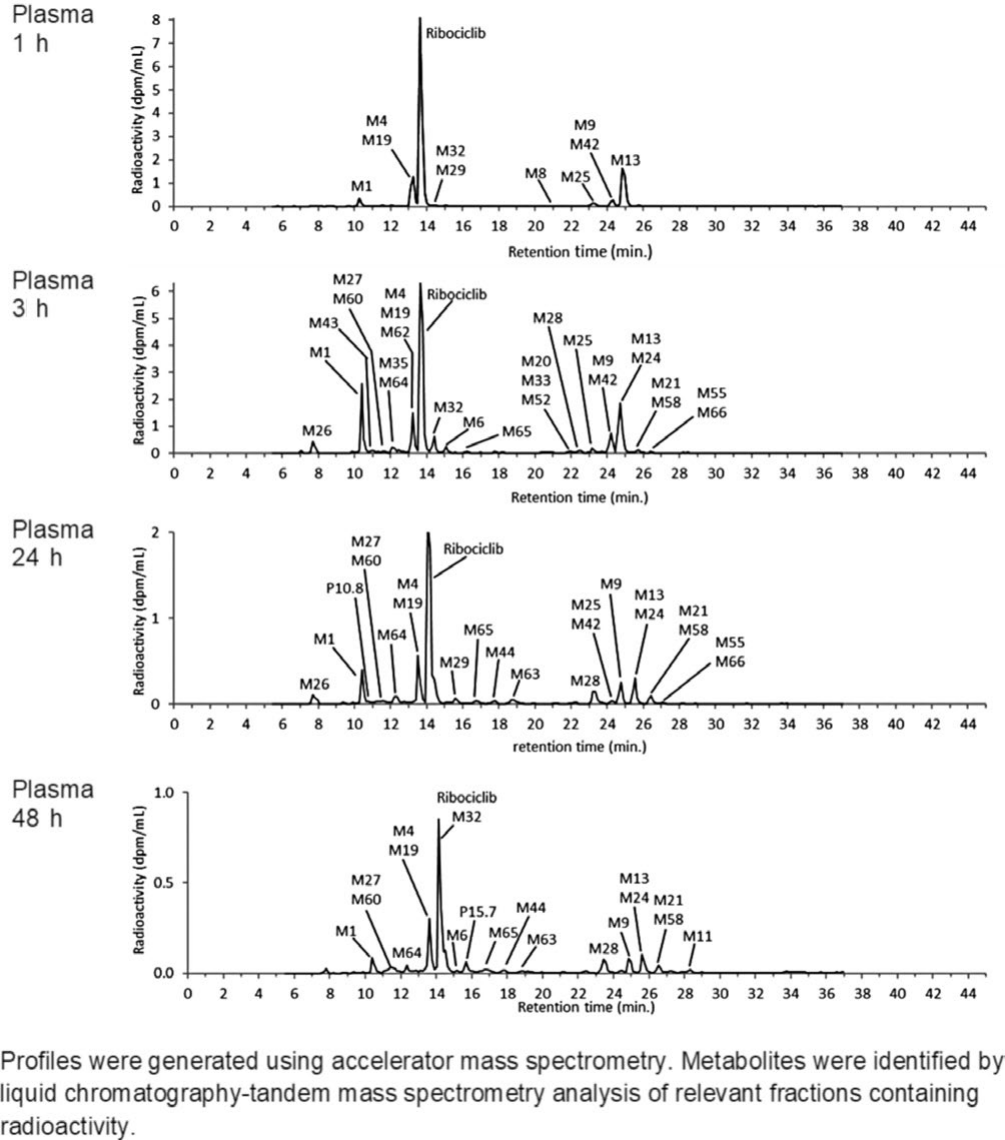
Metabolite patterns in pooled plasma in the human absorption, distribution, metabolism, and elimination study conducted in healthy male volunteers (N = 6) who received a single oral dose of ^14^C‐ribociclib 600 mg. (Y axis units: DPM/mL)

**Figure 5:**
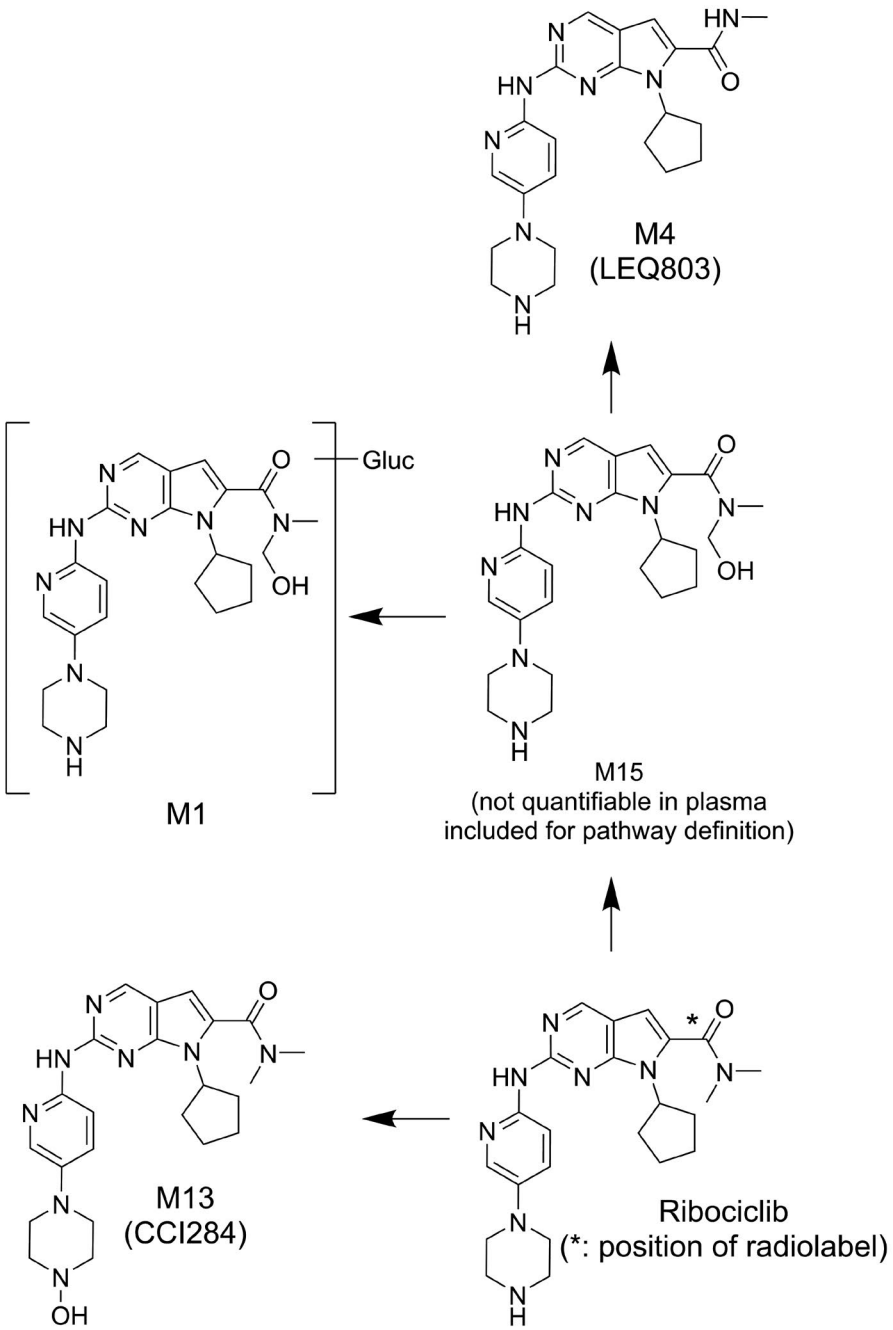
Structures of the most abundant (≥ 7.78% of total drug‐related material AUC0‐48h) circulating (plasma) metabolites of ribociclib in the human absorption, distribution, metabolism, and elimination study conducted in healthy male volunteers (N = 6) who received a single oral dose of ^14^C‐ribociclib 600 mg (a total of 53 metabolites were identified)

Metabolite profiles, generated from plasma pooled across subjects at 1, 3, 24, and 48 hours post dose, are shown in Figure 4. The parent compound was the most abundant component, representing approximately 43.5% of the total radioactivity AUC from time 0 to 48 hours (AUC0‐48) derived from these four time points. At two time points, ribociclib was found to co‐elute with metabolite M32. This metabolite is assumed to have negligible abundance for this assessment, based on comparison of LC‐MS peak areas and assuming similar ionization response. The ratio of AUC0‐48 based on measurements using a validated bioanalytical assay, and AUC0‐48 derived from the metabolite patterns was found to be 0.851. M1 (glucuronidation of M15), M4 (LEQ803, N‐demethylation), and M13 (CCI284, N‐hydroxylation) were the most abundant metabolites in plasma, representing an estimated 7.78, 8.60, and 9.39% of total [^14^C]‐AUC0‐48, respectively (Figure 5; Table S6). M4 was found to co‐elute with metabolites M19 and M62, and M13 with M24 in the plasma patterns. However, the abundance of M24, M19, and M62 is expected to be negligible (≤ 0.475% of total [^14^C]‐AUC0‐48), based on a comparison of LC‐MS peak areas and assuming equivalent LC‐MS ionization of the different metabolites (data on file, Novartis Pharmaceuticals). For metabolite M4, the ratio of AUC0‐48 derived from validated bioanalytical assay data to AUC0‐48 derived from the metabolite patterns was found to be 0.813. Twenty‐nine other metabolites were detected, but all of these were minor in abundance. The largest represented 4.15% (sum of co‐elution of M9 + M42) of total [^14^C]‐AUC0‐48. Notably, metabolite M15 was not observed in plasma, in contrast to previous exploratory studies investigating ribociclib metabolites without the usage of radiolabel (data on file, Novartis Pharmaceuticals). It is known that this metabolite is unstable and degrades to M4. It is likely that this metabolite fully degraded due to the extensive sample preparation needed for AMS measurements. Subsequent LC‐MS analysis of fresh plasma aliquots from this study supported this conclusion, because M15 was detected in these samples although it was not possible to provide a quantitative assessment (data on file, Novartis Pharmaceuticals). Approximately 10.4% of total [^14^C]‐AUC0‐48 was lost during sample preparation procedures.

#### 3.3.7 Metabolite profiles in Excreta

**Figure 6:**
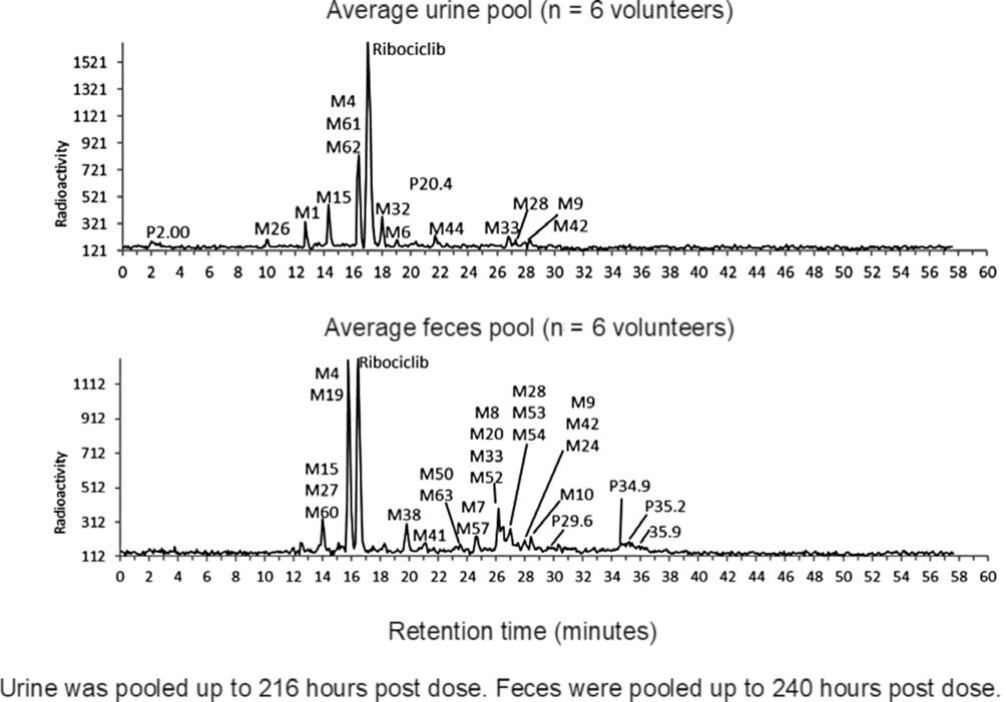
Metabolite patterns in pooled urine and pooled feces in the human absorption, distribution, metabolism, and elimination study conducted in healthy male volunteers (N = 6) who received a single oral dose of ^14^C‐ribociclib 600 mg. (Y axis units: Radioactivity [counts])

A representative metabolite profile of urine and feces samples for the subject pool (N = 6) is shown in Figure 6. Tabulated data showing the percentage of dose attributable to the metabolites is shown in Table S7. The urine pool contained an average of 22.1% of the administered dose (covering 98% of the radioactivity eliminated in urine). The largest drug‐related component identified in urine from radio profiling was ribociclib, representing 12.1% of the dose. Ribociclib in urine was estimated to be 6.75% of the dose by validated bioanalytical assay. The most likely reason for the discrepancy is that minor metabolites in urine under the detection limit of the radio profile led to an overestimation of the ribociclib amount. M4 was also significant, although it was found to co‐elute with metabolites M61 and M62. Assuming that these components were negligible in abundance (based on a comparison of LC‐MS peak areas), M4 represented approximately 3.74% of the administered dose excreted in this matrix. Numerous other metabolites were also detected but these were minor, with each representing ≤ 1.5% of the dose. The feces pool contained an average of 66.8% of the administered dose (covering 97% of the radioactivity eliminated in feces). The largest drug‐related component in feces was ribociclib, representing 17.3% of the administered dose. M4 was also significant, although was found to co‐elute with metabolite M19. Assuming that this metabolite was negligible in abundance (based on a comparison of LC‐MS peak areas), M4 represented approximately 13.9% of the administered dose in this matrix. The next most abundant peak in the radio chromatogram contained four metabolites (M8 [CQM386], M20, M33, and M52) and accounted for 5.21% of the administered dose. Based on an assessment of the individual subject metabolite patterns, the main metabolite in this mixture is either M8 (CQM386) or M33, with the other two being minor. Numerous other metabolites were detected in feces with multiple co‐elutions. However, all of these can be considered minor, with the largest peak in the metabolite pattern accounting for 2.78% of the dose (co‐elution of three metabolites: M28, M53, and M54). Overall, 75.6% of the dose could be assigned to either ribociclib or metabolites in the urine and feces pools, with a further 8.68% of the dose remaining in the feces pellet after extraction. Column recovery was found to be complete in urine, and 92.0% for feces. Considering the exhaustive extraction procedure applied to feces, it is likely that the remaining radioactivity has either extremely strong nonspecific binding to endogenous components in the pellet or is covalently bound.

**Figure 7:**
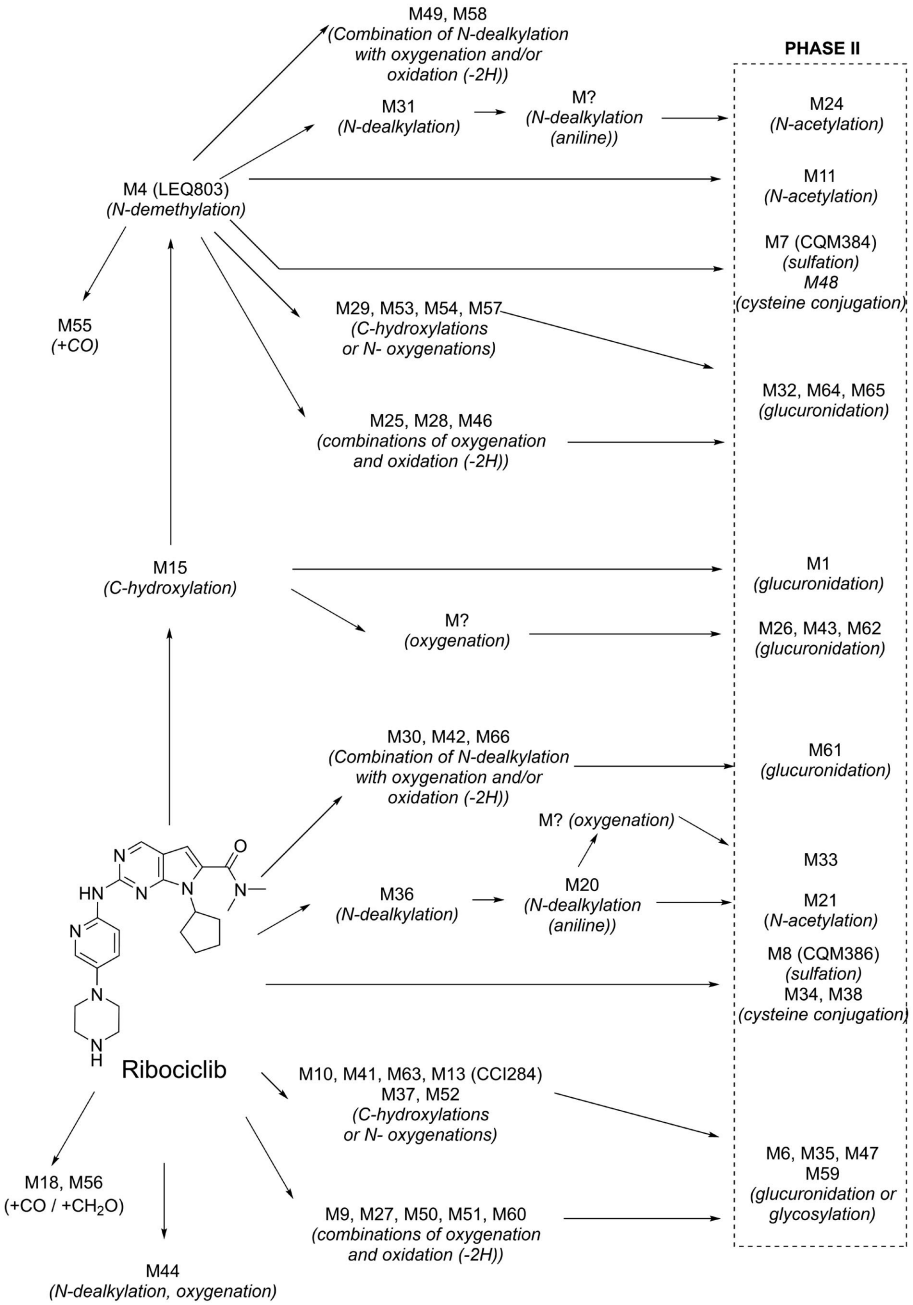
Proposed biotransformations of ribociclib in humans (simplified). Structures are provided in Table S10. M? indicates an intermediate in the pathway which was not observed in the study

The biotransformations in excreta can be summarized as primarily oxidative (dealkylation, C‐ and/or N‐oxygenation, oxidation [−2H]) and combinations thereof. Phase 2 conjugates of Ribociclib Phase 1 metabolites observed in excreta included N‐acetylation, sulfation (M7 [CQM384]), cysteine conjugation, glycosylation, and glucuronidation. Direct Phase 2 conjugates of the parent compound observed were sulfate conjugate M8 (CQM386) and cysteine conjugates M34 and M38. M34 and M38 were minor, with each representing ≤ 2.24% of the dose. The abundance of M8 in excreta could not be reliably estimated due to co‐elutions, but it is < 5.21% of the administered dose. A simplified metabolism scheme is shown in Figure 7, with a summary of MS data and further details on metabolite structures provided in Table S8, Table S9, and Table S10.

## 4 DISCUSSION

### 4.1 Absorption

Ribociclib exhibited moderate to high absorption. In the rat ADME study, absorption (66%) was 1.8‐fold higher than bioavailability (37%), indicating a moderate first pass effect. Bioavailability was higher in dogs (64%‐87%). In the human ADME study, absorption was estimated to be 58.8% based on the mean percentage of the total radiolabeled dose in urine (22.6%) and the amount of dose attributable to metabolites in feces (36.2%), assuming all metabolites in feces were formed systemically and assuming that no ribociclib was excreted into feces via either hepatobiliary export or intestinal secretion. Given that intestinal secretion is suspected based on rat ADME data, this absorption value in human should be considered with caution. Oral bioavailability could not be calculated in the human ADME study as co‐administration of a ^13^C‐iv microdose was not conducted.

### 4.2 Distribution

In tissue distribution studies in male rats, total radiolabeled components were markedly distributed into the extravascular compartment except for brain and were eliminated rapidly from most tissues. In pigmented rats, specific distribution of radioactivity to the melanin‐containing structures was observed. Ribociclib was found to pass the placental barrier in both rats and rabbits, and was excreted into the milk of lactating rats. Although placental transfer and milk excretion was observed in animals, it should be acknowledged that translatability of the data to human is complex and not well understood due to anatomical and functional differences of the placenta between species, and wide species differences in the protein and lipid content of milk.^19^, ^20^, ^21^ Based on population PK analysis^22^ (Lu et al, manuscript submitted), ribociclib was also extensively distributed in humans, with an apparent volume of distribution at steady state of 1090 L (total of the estimated central and peripheral volume of distribution). Plasma protein binding showed up to 1.7‐fold difference between species, with fraction unbound in human being 0.30.

### 4.3 Metabolism

Following oral administration of a single 600 mg dose of [^14^C]‐ribociclib to healthy male volunteers, the primary metabolic pathways involved oxidation (dealkylation, C‐ and/or N‐oxygenation, oxidation (−2H)) and combinations thereof. Phase II conjugates of ribociclib phase I metabolites were also observed, such as N‐acetylation, sulfation, cysteine conjugation, glycosylation, and glucuronidation. Ribociclib was the major circulating component in plasma, representing 43.5% of the total radioactivity AUC0‐48 based on metabolite profiling of four time points. However, the overall contribution of ribociclib to total radioactivity in plasma based on AUCinf was 23%. The difference is likely due to the long half‐life of total radioactivity (293 h) compared to ribociclib (54.7 h) (Table 3), which could be caused by slow elimination of low concentrations (below the LLOQ) of ribociclib or metabolites, or covalent binding of drug‐related material to endogenous plasma components. The presence of cysteine adducts (M34, M38, and M48) in the human excreta profiles suggests the possible formation of reactive intermediates. However, these metabolites were present in low abundance with only M38 being quantifiable in the n = 6 subject feces pool, suggesting that this pathway is minor. The main plasma metabolites were M1 (glucuronidation of M15), M4 (LEQ803, N‐demethylation), and M13 (CCI284, N‐hydroxylation), which represented 7.78, 8.60, and 9.39% of total radioactivity (Table S6), and 17.9, 19.8, and 21.6% of ribociclib exposure, respectively. Twenty‐nine other metabolites were identified in plasma, but these were minor (≤4.15%). Subsequent investigations, by bioanalytical assay or by relative exposure comparison across species,^23^, ^24^ confirmed that metabolites M4 and M13 were covered by rat, dog, and/or rabbit (data on file, Novartis Pharmaceuticals). Furthermore, M4 and M13 were found not to have a relevant contribution to total pharmacological activity in human considering both in vitro measurements and their in vivo exposure.

### 4.4 Excretion

Ribociclib is mainly eliminated via metabolic clearance, with renal clearance playing a lesser role. Median renal clearance (CLr) of ribociclib was more than 10‐fold lower than the non‐renal clearance (5.55 L/h vs 64.7 L/h). The majority of the total radiolabeled dose was excreted in the feces (69.1%), with 22.6% found in urine (Figure 3). This is similar to preclinical species, where 68.8%‐84% and 5.9%‐18.5% of administered radioactivity were found in feces and urine, respectively. Elimination occurred mainly by hepatic metabolism with a limited contribution of renal excretion of unchanged ribociclib, which represented 6.75% of the dose in urine, measured by validated bioanalytical assay (17.3% of dose in feces). A large number of metabolites were identified in excreta (Figure 6, Table S7), but the most abundant was M4 (LEQ803), which represented 13.9 and 3.74% of the dose in feces and urine, respectively.

### 4.5 Enzyme phenotyping

Using recombinant human enzymes, metabolites M4 (LEQ803) and M15 were efficiently formed by CYP3A4, but were almost not detectable in incubations of the other hepatic CYPs investigated. A correlation analysis (Table S3) also strongly suggested that the enzymatic formation of M4, M15, and minor metabolites is catalyzed by CYP3A.

The hydroxylamine metabolite M13 (CCI284) was formed readily by FMO3 and CYP2J2, but not by the other recombinant hepatic CYPs. Inactivation of FMO3 by heat treatment, correlation analysis (Table S3), and incubation with recombinant enzymes consistently indicated that hydroxylamine M13 (CCI284) is formed by FMO3.

Based on the kinetics of metabolite formation (Figure 2), it was estimated that 74% of the oxidative metabolism in HLM results from CYP3A4, whereas 26% is due to the hepatic FMO contribution. Incubation with CYP‐selective inhibitors suggests a dominant contribution of CYP3A4/5 to oxidative metabolism in HLM (Table 2).

Collectively, all in vitro enzyme phenotyping approaches consistently indicate that oxidative hepatic metabolism of ribociclib is dominated by CYP3A4/5 (74%) with partial contribution by FMO3 (15%‐26%). It is likely that inhibitors and/or inducers of CYP3A4 will influence the oxidative metabolic clearance of ribociclib in humans.

### 4.6 Estimation of ribociclib elimination pathways

**Figure 8:**
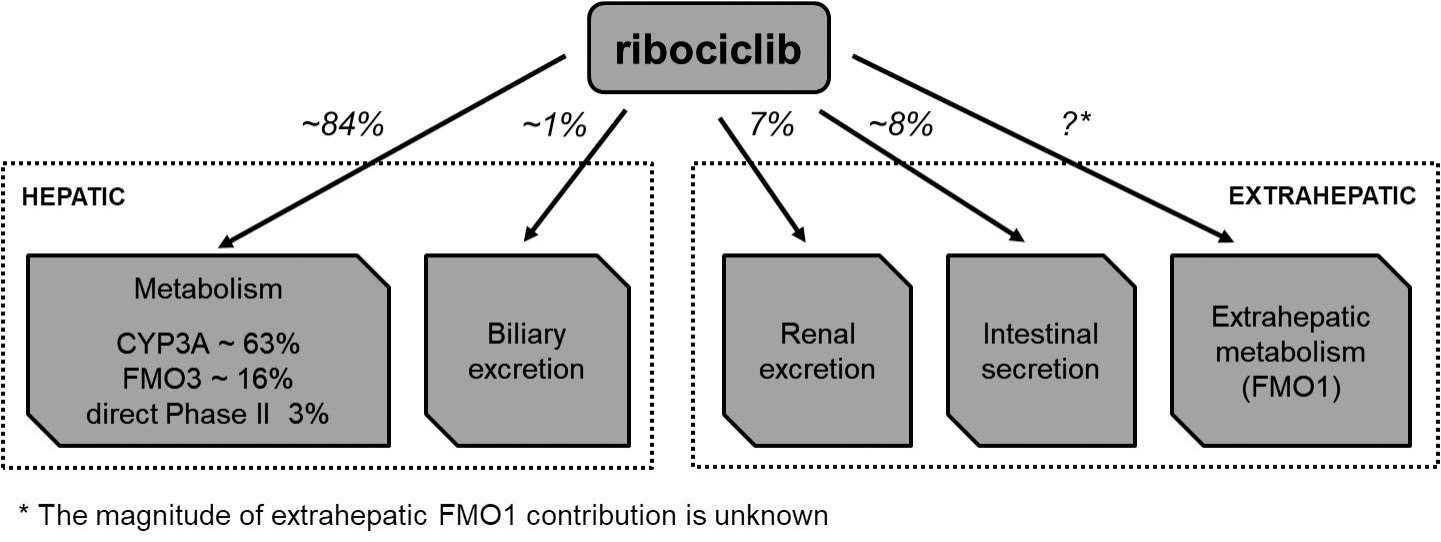
Schematic description of anticipated ribociclib elimination pathways based on results of absorption, distribution, metabolism, and elimination studies in humans (renal excretion) and rats (biliary excretion, intestinal secretion) and in vitro enzyme phenotyping

Integrating the human ADME results obtained in healthy male volunteers, the metabolic enzyme phenotyping, and rat biliary and intestinal excretion data, our data indicate that ribociclib is eliminated mainly by hepatic metabolism in humans, primarily via CYP3A with a lesser contribution by FMO3 and direct phase II metabolism (approx. 84% of total). The remainder is accounted for by renal excretion (7%), intestinal excretion (8%), biliary elimination (1%), and an unknown, but likely low, contribution from extrahepatic metabolism (FMO1) (Figure 8). The intestinal excretion and biliary elimination components are based on rat ADME data, and assume that these data translate to human.

## DISCLOSURES

James, Alexander David; Schiller, Hilmar; Marvalin, Cyrille; Jin, Yi; Borell, Hubert; Glaenzel, Ulrike; Ji, Yan and Camenisch, Gian are employees of Novartis and may own shares in Novartis.

## AUTHOR CONTRIBUTIONS

James, Schiller, Marvalin, Jin, Glaenzel (human ADME), Roffel, and Camenisch participated in research design*.* James, Schiller, Marvalin, Jin, Borell conducted experiments. James, Schiller, Marvalin, Jin, Borell, and Roffel performed data analysis. James, Schiller, Marvalin, Jin, Borell, Roffel, and Ji wrote or contributed to the writing of the manuscript. All authors have contributed to the manuscript and submitted the study.

## Supporting information

Supplementary Material
