# Supplementary material for: An integrated assessment of the ADME properties of the CDK4/6 Inhibitor ribociclib utilizing preclinical in vitro, in vivo, and human ADME data
Source: Pharmacol Res Perspect. 2020 Jun 10;8(3):e00599. doi: 10.1002/prp2.599 (PMC7287031; doi:10.1002/prp2.599)
Supplement: Supplementary file 1 — Supplementary Material [file PRP2-8-e00599-s001.docx]

**Title: An integrated assessment of the ADME properties of the CDK4/6 Inhibitor ribociclib utilizing preclinical in vitro, in vivo and human ADME data.**

**Authors:** James, Alexander David; Schiller, Hilmar; Marvalin, Cyrille; Jin, Yi; Borell, Hubert, Roffel, Ad F; Glaenzel, Ulrike; Ji, Yan; Camenisch, Gian.

PK Sciences (ADME), Novartis Institutes for Biomedical Research, Basel, Switzerland. (ADJ, HS, CM, YJ, HB, UG, GC)

PK Sciences (Oncology TA), Novartis Institutes for Biomedical Research, East Hanover, USA (YJ)

PRA Health Sciences, Scientific and Medical Affairs, Groningen, the Netherlands. (AFR)

**Journal title:** Pharmacology Research and Perspectives

**Supporting information figure legends:**

**Figure S1:** Radio chromatogram of incubates of [^3^H] ribociclib with human liver microsomes in the presence of NADPH

**Figure S2:** Enzyme kinetics of [^3^H] ribociclib in HLM

**Figure S3:** Metabolite profiles in male and female rat plasma and excreta after [^3^H] ribociclib I.V. dosing

**Figure S4:** Metabolite profiles in male dog plasma and excreta after [^3^H] ribociclib I.V. dosing

**Supporting information table legends:**

**Table S1**: Experimental conditions of pre-clinical pharmacokinetic and ADME studies

**Table S2:** Mean pharmacokinetic parameters of ribociclib in plasma of animals after single-dose intravenous or oral administration

**Table S3:** Correlation of ribociclib metabolite formation with marker enzyme activities in a bank of single donor human liver microsomes

**Table S4:** Synthesis schemes for the preparation of [^14^C] and [^3^H] - ribociclib

**Table S5**: Calculation of fraction of compound in plasma (fp) and blood to plasma concentration ratio (Cbc/Cp)

**Table S6**: Ribociclib and metabolites in plasma pools (n=6)

**Table S7**: Ribociclib and metabolites in excreta pools (n=6)

**Table S8**: Summary of LC-MS/MS data of [^14^C] ribociclib and metabolites observed in plasma, urine and feces

**Table S9**: Summary of LC-MS exact mass and hydrogen/deuterium exchange data of [^14^C] ribociclib and metabolites observed in plasma, urine and feces

**Table S10**: Structures of ribociclib and its metabolites identified in human

**Table S11**: Cumulative total radiolabeled components excreted in urine and feces

**Figure S1: Radio chromatogram of incubates of [^3^H] ribociclib with human liver** **microsomes in the presence of NADPH.**

1. 5 µM [^3^H] ribociclib, 0.4 mg protein/mL HLM, 15 min

1. 5 µM [^3^H] ribociclib, 0.4 mg protein/mL of HLM treated 1 min at 50°C (FMO inactivation), 15 min

**Retention time (min)**

**Figure S2: Enzyme kinetics of [^3^H] ribociclib in HLM**

The concentration-dependent kinetics of [^3^H] ribociclib biotransformation (total metabolism formation) in pooled HLM (0.4 mg/mL) after 8 min or 15 min incubation was fitted with the Michaelis-Menten equation and plotted as Michaelis-Menten plot (upper) and as Eadie-Hofstee plot (lower) using nominal substrate concentrations of 0.125 µM to 300 µM.

**Figure S3: Metabolite profiles in male and female rat plasma and excreta after [^3^H] ribociclib i.v. dosing.**

Radio chromatograms of plasma (2 h and 8 h) and excreta (0-72 h) from male HAN:WIST rats after a single i.v. dose of 4 mg/kg [^3^H] ribociclib (pools of three rats)

Radio chromatograms of plasma (2h and 8 h) and excreta (0-72 h) from female HAN:WIST rats after a single i.v. dose of 4 mg/kg [^3^H] ribociclib (pools of three rats)

**Figure S4: Metabolite profiles in male dog plasma and excreta after [^14^C] ribociclib p.o. dosing.**

Radio chromatograms of plasma and excreta from male dogs after a single p.o. Dose of 5 mg/kg [^14^C] ribociclib (pools of three dogs)

**Table S1. Experimental conditions of pre-clinical pharmacokinetic and ADME studies**

| Study type | Animals | Test compounds | Doses | Vehicle | Performed analysis |
| --- | --- | --- | --- | --- | --- |
| Dog PK study | Beagle dog, male (n=3 per dosing route) | Ribociclib free base | 0.2 mg/kg (iv) and 0.5 mg/kg (po) | iv: solution in tartaric acid (0.2 M) / propylene glycol / dextrose (5% in water) (1:10:89 v/v/v)  po: solution in tartaric acid (0.2 M) / propylene glycol (1:10 v/v) | **Kinetics:** 0.083 (i.v. group only), 0.25, 0.5, 1, 2, 4, 6, 8 and 24 h post-dose |
| Rat PK study | Sprague Dawley rats, male (n=2 (iv), n=3 (po) | Ribociclib free base | 2 mg/kg (iv) and 5 mg/kg (po) | iv and po: solution in 10% 0.1N HCl / 20% propylene glycol / 10% chremophor / 60% 50 mM acetate buffer pH 4.63 | **Kinetics:** 0.083 (i.v. group only), 0.25, 0.5, 1, 2, 4 and 8 h post-dose |
| Rat ADME | Rats, Hanover Wistar, albino, males  Rats, Lewis, pigmented, males (for QWBA) | [^3^H]Ribociclib | 4 mg/kg (iv) and 10 mg/kg (po) | iv : 5% aqueous glucose solution  po: 0.5% aqueous methyl cellulose solution | **Kinetics:** (iv, 3 rats) (po, 3 rats), blood/plasma TP (min): 0.083 (only iv), 0.25, 0.5, 1, 2, 4, 8, 24, 32, 48, 72, 96, 168.  **Excretion/metabolism**: (iv, 3 rats) (po, 3 rats), blood/plasma TP (min): 0.083 (only iv), 0.25 (only po), 2, 4, 8, 24, 48, 72, 96, 168. excreta: daily up to 96h and 96-168h  **QWBA:** (iv, 2 rats Lewis, 24-168h), (iv, 4 rats HW, 0.083, 1, 24, 168h), (po, 6 rats HW, 0.25, 2, 8, 24, 72, 168)  **Bile excretion:** iv, 4 rats; bile TP : 0-24h and 24-48h ; urine/feces: daily up to 48h |
| Rat ADME | Rats, Hanover Wistar, albino, females | [^3^H]Ribociclib | 4 mg/kg (iv) | 5% aqueous glucose solution | **Kinetics:** 3 rats, blood/plasma TP (min): 0.083, 0.25, 0.5, 1, 2, 4, 8, 24.  **Excretion/metabolism**: 3 rats, blood/plasma TP (min): 0.083, 2, 4, 8, 24. excreta: daily up to 72h |
| Dog PK study | Beagle dog, male (n=4 per treatment) | Ribociclib succinate, metabolite LEQ803 | 200 mg/dog (mg free base) | Treatment A: hard gelatin capsule with ribociclib succinate and excipients Treatment B: hard gelatin capsule with neat ribociclib succinate Treatment C: ribociclib succinate solution in water | **Kinetics**: 0 (pre-dose), 0.25, 0.5, 1, 2, 4, 7, 10, 24, 48 h post-dose |
| Dog ADME | 3 Beagle dogs, males | [^14^C]Ribociclib | 5 mg/kg (po) | 0.5% aqueous methyl cellulose solution | **Blood/plasma:** Pre-dose, 0.25, 0.5, 1, 2, 4, 8, 12, 24, 32, 48, 72, 96, 120, 168, 312, 360, 408, 504.  **Urine, feces, cage wash:** daily up to 168h and at selected intervals up to 1152h |
| Rat embryofetal transfer | Rats, Hanover Wistar, albino, pregnant females (n=5 for toxicokinetics) | Ribociclib succinate | 50, 300, 1000 mg/kg/day (po) on days 6 to 17 postcoitum | 0.5% aqueous methyl cellulose solution | **Maternal blood/plasma:** 0.5, 1, 3, 7 and 24h post-dose on day 16 postcoitum  **Fetal blood/plasma**: 3 h post dosing on day 17 postcoitum |
| Rabbit embryofetal transfer | Rabbits, New Zealand White, pregnant females (n=5 for toxicokinetics) | Ribociclib succinate | 10, 30, 60 mg/kg/day (po) on days 7 to 20 postcoitum | 0.5% aqueous methyl cellulose solution | **Maternal blood/plasma:** 0.5, 1, 3, 7 and 24h post-dose on day 19 postcoitum  **Fetal blood/plasma**: 3 h post dosing on day 20 postcoitum |
| Rat ADME (milk excretion) | Rats, Hanover Wistar, albino, lactating females | [^14^C]Ribociclib | 50 mg/kg (po) | 0.5% aqueous methyl cellulose solution | **Blood and milk:** Group 1 (n=4): TP (h) 0.25, 4, 24, 72. Group 2 (n=4): TP (h) pre-dose, 1, 8, 48. |
| Rat QWBA | Rats, Long Evans, partially pigmented, females | [^3^H]Ribociclib | 10 mg/kg (po) | 0.5% aqueous methyl cellulose solution | **Blood/plasma:** for total RA and Ribociclib: 3 rats TP (h): 0.25, 0.5, 1, 2, 4, 8, 24, 32, 48, 96, 168.  **Blood/plasma (for total RA) + QWBA:** 6 rats (1/TP), TP (h): 1, 4, 8, 24, 168, 336. |

**Table S2. Mean pharmacokinetic parameters of ribociclib in plasma of animals after single-dose intravenous or oral administration**

| **Species** | **Sex, (n)** | **Dosing route** | **Dose (mg base/kg)** | **t_max_ (h)** | **C_max_ (ng/mL)** | **AUC_last_ (ng·h/mL)** | **AUC interval  (h)** | **t_½_ (h)** | **CL**  **(L/h/kg)** | **V_ss_**  **(L/kg)** | **F** |
| --- | --- | --- | --- | --- | --- | --- | --- | --- | --- | --- | --- |
| Rat | m (2) | i.v. | 2.0 | - | - | 586.6 | 8 | 2.8 | 3.1 | 9.9 | - |
| Rat ^1)^ | m (3) | i.v. | 4.1 | - | - | 1442.7 | 8 | 3.2 | 2.8 | 7.9 | - |
| Rat | f (3) | i.v. | 4.2 | - | - | 508.4 | 8 | 1.9 | 7.8 | 12.2 | - |
| Dog | m (3) | i.v. | 0.2 | - | - | 105.2 | 24 | 18.1 | 1.9 | 27.9 | - |
| Rat | m (3) | po | 5.0 | 2.0 | 126.0 | 672.0 | 8 | - | - | - | 55% |
| Rat ^1)^ | m (3) | po | 9.8 | 4.0 | 130.4 | 1338.0 | 24 | - | - | - | 37% |
| Rat | f (3) | po | 11.1 | 2.0 | 59.1 | 359.8 | 24 | - | - | - | - |
| Rat | f (4) | po | 51.2 | 0.3 | 1086.0 | 3098.0 | 8 | 2.0 | - | - | - |
| Dog | m (3) | po | 0.5 | 3.0 | 10.4 | 155.1 | 24 | - | - | - | 64% |
| Dog | m (3) | po | 5.0 | 1.0 | 205.5 | 4519.0 | 168 | 38.8 | - | - | - |
| Dog | m (4) | po | 18.9 | 1.0 | 1220.0 | 21100.0 | 48 | 12.6 | - | - | 87% |

AUClast, area under the concentration–time curve from time 0 to time of last measurable concentration; CL, plasma clearance; Cmax, maximum concentration; F, bioavailability; f, female; i.v., intravenous; m, male; po, oral; t½, elimination half-life; tmax, time to maximum concentration; V_SS_, volume of distribution at steady state. ^1)^ Absorption estimated at 66%, derived from AUC of total radiolabeled components after i.v. and p.o. dosing (data on file, Novartis pharmaceuticals).

**Table S3. Correlation of ribociclib metabolite formation with marker enzyme activities in a bank of single donor human liver microsomes**

A bank of individual human liver microsomes (n=16) was incubated with 20 µM [^3^H] ribociclib. The enzymatic rates of different metabolic pathways were correlated with known activity of enzymes in the same bank of HLM. Data shown are linear regression coefficients (R).

|  |  | **Linear regression coefficient (R)** | | | | |
| --- | --- | --- | --- | --- | --- | --- |
| Marker reaction | Enzyme | M15 | M4 (LEQ803) | CCI284 | minor metabolites | total metabolism |
| 7-Ethoxyresorufin O-dealkylation | CYP1A2 | 0.038 | 0.053 | 0.444 | 0.121 | 0.124 |
| Phenacetin O-deethylation | CYP1A2 | 0.217 | 0.227 | 0.364 | 0.321 | 0.292 |
| Coumarin 7-hydroxylation | CYP2A6 | 0.221 | 0.258 | -0.318 | 0.254 | 0.196 |
| S-Mephenytoin N-demethylation | CYP2B6 | 0.508 | 0.506 | -0.167 | 0.538 | 0.481 |
| Bupropion hydroxylation | CYP2B6 | 0.256 | 0.224 | -0.160 | 0.264 | 0.218 |
| Paclitaxel 6α-hydroxylation | CYP2C8 | 0.471 | 0.512 | 0.303 | 0.544 | 0.537 |
| Diclofenac 4´-hydroxylation | CYP2C9 | 0.184 | 0.253 | 0.026 | 0.226 | 0.222 |
| S-Mephenytoin 4´-hydroxylation | CYP2C19 | 0.211 | 0.210 | -0.145 | 0.177 | 0.178 |
| Dextromethorphan O-demethylation | CYP2D6 | 0.400 | 0.427 | -0.045 | 0.427 | 0.403 |
| Chlorzoxazone 6-hydroxylation | CYP2E1 | 0.102 | 0.074 | 0.139 | 0.172 | 0.125 |
| Testosterone 6β-hydroxylation | CYP3A4/5 | **0.977** | **0.988** | 0.101 | **0.981** | **0.975** |
| Midazolam 1-hydroxylation | CYP3A4/5 | **0.865** | **0.884** | 0.177 | **0.925** | **0.893** |
| Lauric acid 12-hydroxylation | CYP4A11 | -0.016 | 0.029 | 0.663 | 0.033 | 0.104 |
| Benzydamine N-Oxidation | FMO | -0.086 | -0.104 | **0.764** | -0.047 | 0.022 |

**Table S4. Synthesis schemes for the preparation of [^14^C] and [^3^H]- ribociclib**

| [^14^C]-Ribociclib | |
| --- | --- |
| Reaction Conditions:  1) TMS-acetylene, CuI, Pd(Ph)_2_Cl_2_, Et_3_N, 55^o^C in DMF (44%)  2) NIS, AgNO_3_, DMF (81%)  3) 1M TBAF in THF, THF (50%)  4) *n*-BuLi, Et_2_O, -90^o^C then ^14^CO_2_, -78^o^C (89%)  5) CDI, THF then 2M HNMe_2_ in THF (81%)  6) A2, Pd(OAc)_2_, BINAP, K_2_CO_3_, MIBK, 100oC (77%)  7) 6M HCl, MIBK (>99%)  8) succinic acid, IPA, 80^o^C (88%) | 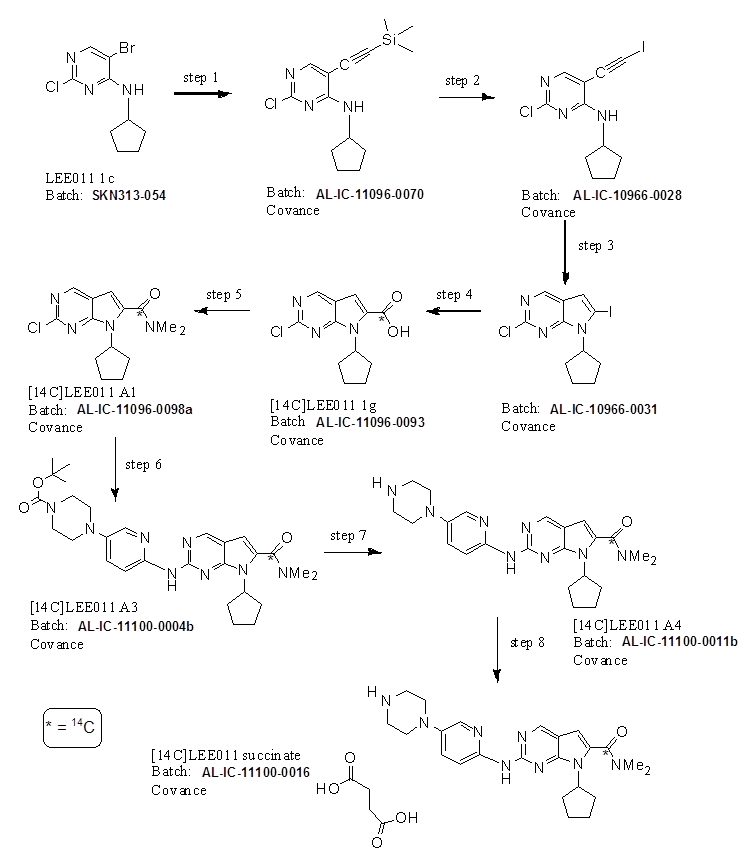 |
| [^3^H]-Ribociclib | |
| Iodinated ribociclib was tritiated by reacting it with ^3^H_2_, in the presence of palladium on charcoal and triethylamine in DMF. The resulting product was purified by preparative HPLC, diluted with unlabelled ribociclib to afford final [^3^H]-ribociclib with the required radiochemical purity of 98.6% and a specific activity of 108.1MBq/mg |  |

**Table S5. Calculation of fraction of compound in plasma (fp) and blood to plasma concentration ratio (Cbc/Cp)**

| The fraction of compound in plasma (fp) was calculated as follows:  Fp(%) = (Cp/Cb) x (1-H) x 100  Where Cb is the concentration in blood, Cp is the concentration in plasma and H is the hematocrit value.  Standard deviation (SD) of fp was calculated using formula for error propagation for divisions as follows assuming the hematocrit value as a constant.  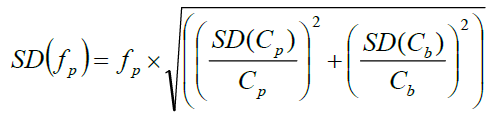  The concentration in blood cells (Cbc) was calculated as follows:  Cbc = [(Cb-Cp) x (1-H)] /H  and used for the calculation of the blood cells to plasma concentration ratio (Cbc/Cp). |
| --- |

**Table S6. Ribociclib and metabolites in plasma pools (n=6)**

Exposure of ribociclib and metabolites in plasma (individual time point pools of n=6 healthy subjects), following a single oral dose of 600 mg [^14^C] ribociclib; data derived from metabolite patterns; components listed in order of elution; where co-elutions exist, components listed in order of LC-MS retention time where possible. Due to the complex co-elution pattern that occasionally differed between individual time points, Individual components were grouped when required in order to estimate AUC.

| Sample collection time (h) | 1 | 3 | 24 | 48 | 0-48 | |
| --- | --- | --- | --- | --- | --- | --- |
|  | Concentration | | | | AUC0-48h | |
| Component | nM | | | | nM*h | % |
| M26 | - | 50.9 | 14.6 | - | 914 | 1.91 |
| M1 | 42.3 | 230 | 37.9 | 14.0 | 3730 | 7.78 |
| P10.8 | - | - | 4.34 | - | 97.7 | 0.204 |
| M43 | - | 19.9 | - | - | 229 | 0.478 |
| M27+M60 | - | 16.0 | 11.4 | 12.0 | 584 | 1.22 |
| M35 + M64 | - | 51.9 | 15.3 | 10.3 | 1070 | 2.22 |
| M4+M19+M62 | 219 | 143 | 68.8 | 50.7 | 4130 | 8.60 |
| Ribociclib+M32 | 1150 | 847 | 328 | 168 | 20900 | 43.5 |
| M32+M29 | 17.0 | 73.3 | 9.28 | - | 1080 | 2.25 |
| M6 | - | 28.0 | - | 2.00 | 346 | 0.721 |
| P15.7 | - | - | - | 12.7 | 152 | 0.316 |
| M65 | - | 12.7 | 6.84 | 6.34 | 375 | 0.783 |
| M44 | - | - | 5.50 | 2.33 | 152 | 0.316 |
| M63 | - | - | 11.8 | 1.90 | 288 | 0.599 |
| M8 (CQM386) | 9.50 | - | - | - | 14.2 | 0.0297 |
| M20+M33+M52 | - | 11.9 | - | - | 137 | 0.286 |
| M28 | - | 17.7 | 26.9 | 16.9 | 1010 | 2.11 |
| M25+M42 | 33.7 | 25.9 | 5.43 | - | 470 | 0.980 |
| M9+M42 | 47.5 | 93.4 | 29.3 | 15.6 | 1990 | 4.15 |
| M13 (CCI284)+M24 | 316 | 256 | 37.8 | 19.1 | 4500 | 9.39 |
| M21+M58 | - | 22.0 | 13.6 | 8.43 | 660 | 1.38 |
| M55+M66 | - | 10.0 | 3.27 | - | 189 | 0.394 |
| M11 | - | - | - | 2.34 | 28.1 | 0.0585 |
| Sum of additional components | - | - | - | - | - | - |
| Total Detected | 1830 | 1910 | 630 | 342 | 43000 | 89.6 |
| Lost during sample processing | 206 | 280 | 55.6 | 15.4 | 4970 | 10.4 |
| Lost during HPLC | - | - | - | - | - | - |
| Total radiolabeled components in original sample | 2040 | 2190 | 686 | 358 | 48000 | 100 |

**Table S7**: **Ribociclib and metabolites in excreta pools (n=6)**

Mean amounts of ribociclib and metabolites in the pooled excreta of six healthy male human subjects, following a single oral dose of 600 mg [^14^C] ribociclib; data derived from metabolite patterns; components listed in order of elution; where co-elutions exist, components listed in order of LC-MS retention time where possible

| Components | Excretion (% of dose) | | |
| --- | --- | --- | --- |
|  | Urine | Feces | Total |
| P2.0 | 0.273 | - | 0.273 |
| M26 | 0.252 | - | 0.252 |
| M1 | 0.642 | - | 0.642 |
| M27 + M60 + M15 | - | 2.28 | 2.28 |
| M15 | 1.50 | - | 1.50 |
| M19 + M4 (LEQ803) | - | 13.9 | 13.9 |
| M62 + M61 + M4 (LEQ803) | 3.74 | - | 3.74 |
| Ribociclib | 12.1 | 17.3 | 29.4 |
| M32 | 1.24 | - | 1.24 |
| M6 | 0.316 | - | 0.316 |
| P20.4 | 0.397 | - | 0.397 |
| M38 | - | 2.24 | 2.24 |
| M44 | 0.591 | - | 0.591 |
| M41 | - | 1.10 | 1.10 |
| M63 + M50 | - | 0.829 | 0.829 |
| M7 (CQM384) + M57 | - | 1.37 | 1.37 |
| M20 + M8 (CQM386) + M33 + M52 | - | 5.21 | 5.21 |
| M33 | 0.479 | - | 0.479 |
| M53 + M28 + M54 | - | 2.78 | 2.78 |
| M28 | 0.264 | - | 0.264 |
| M42 + M9 | 0.276 | - | 0.276 |
| M42 + M9 + M24 | - | 0.975 | 0.975 |
| M10 | - | 1.14 | 1.14 |
| P29.6 | - | 0.676 | 0.676 |
| P34.9 | - | 1.32 | 1.32 |
| P35.2 | - | 1.46 | 1.46 |
| P35.9 | - | 0.918 | 0.918 |
| Sum of additional components | - | - | - |
| Total detected | 22.1 | 53.5 | 75.6 |
| Lost during sample processing | - | 8.68 | 8.68 |
| Lost during HPLC | - | 4.65 | 4.65 |
| RA in sample pool (% of dose) | 22.1 | 66.8 | 88.9 |

**Table S8. Summary of LC-MS/MS data of [^14^C] ribociclib and metabolites observed in plasma, urine and feces**

Data from LC-MS/MS runs of plasma, urine and feces from healthy male subjects following a single oral dose of 600 mg [^14^C] ribociclib. Metabolites listed in numerical order, proposed structures are shown in Table S10.

| Component | Matrix | Observed ions in LC-(MS/MS) runs (*m/z*) ^a)^ | | | | | |
| --- | --- | --- | --- | --- | --- | --- | --- |
|  |  | [M+H]^+^ | A | B | C | D | Additional major signals |
| Ribociclib | p, u, f | 435 | 367 | 322 | 294 | 252 | 162 |
| M1 | p, u | 627 | - | 322 | 294 | - | 451, 421, 390, 353 |
| M4 (LEQ803) | p, u, f | 421 | 353 | 322 | 294 | 252 | - |
| M6 | p, u | 627 | 383 | 338 | - | - | 451 |
| M7 (CQM384) | f | 501 | 353 | 322 | 294 | 252 | 421 |
| M8 (CQM386) | p, f | 515 | 367 | 322 | 294 | 252 | 435 |
| M9 | p, u, f | 449 | 381 | 336 | 308 | - | 176 |
| M10 | f | 451 | 383 | 338 | 310 | - | 321, 253, 251, 238, 205, 178 |
| M11 | p, f | 463 | 395 | 364 | 336 | - | 322, 294, 231, 204, 189, 162 |
| M13 (CCI284) | p | 451 | 383 | 338 | 310 | - | 392 |
| M15 | u, f | 451 | - | 322 | 294 | 252 | 421, 353 |
| M18 | f | 463 | 395 | 350 | 322 | - | 294, 217, 190 |
| M19 | p, f | 409 | 341 | 296 | 268 | - | 279, 251, 238, 163, 136, 107 |
| M20 | p, u, f | 366 | - | 253 | 225 | - | 198 |
| M21 | p, f | 408 | - | 295 | 267 | - | 240, 225, 208, 162, 135, 120 |
| M24 | p, f | 394 | - | 295 | 267 | - | 240, 225, 208, 162, 135, 120 |
| M25 | p | 437 | 369 | 338 | 310 | - | 378 |
| M26 | p, u | 643 | 365 | - | 294 | - | 467, 449, 437, 419, 406, 388, 360, 353, 340, 322, 294 |
| M27 | p, f | 433 | 365 | 320 | 292 | 250 | - |
| M28 | p, u, f | 435 | 367 | 336 | 308 | - | - |
| M29 | p | 437 | 369 | - | - | - | 324, 296 |
| M30 | f | 437 | - | - | - | - | 369, 324, 296, 238, 191, 164 |
| M31 | f | 395 | 327 | 296 | 268 | - | 251, 238 |
| M32 | p, u | 613 | 369 | 338 | 310 | - | 437, 378 |
| M33 | p, u, f | 424 | - | 311 | 283 | - | - |
| M34 | f | 554 | 486 | 441 | - | - | 467, 450, 434, 399, 382, 354, 337 |
| M35 | p | 627 | 383 | 338 | - | - | 451, 408, 295 |
| M37 | f | 467 | 399 | 354 | 326 | - | 409, 341, 308, 296 |
| M38 | f | 554 | 486 | 441 | - | - | 537, 493, 467, 465, 434, 424, 399, 392, 380, 354, 352, 326, 324 |
| M41 | u, f | 451 | 383 | 338 | 310 | - | 408, 321, 295, 281, 267, 255 |
| M42 | p, u, f | 410 | 342 | 297 | 269 | - | 238 |
| M43 | p, u | 643 | 381 | 338 | 310 | - | 467, 437, 406, 378, 369 |
| M44 | p, u | 290 | - | 177 | 149 | - | 160, 132 |
| M46 | f | 451 | 367 | 336 | 308 | - | 433, 281, 203, 176 |
| M47 | f | 641 | 573 397 | 528 352 | 500 324 | - | 613, 595, 465, 307 |
| M48 | f | 540 | 472 | 441 | - | - | 523, 479, 453, 420, 385, 354 |
| M49 | f | 423 | 355 | 324 | 296 | - | 310, 282, 265, 210, 164 |
| M50 | f | 463 | 395 | 350 | 322 | - | 294, 217, 190 |
| M51 | f | 481 | - | 324 | 296 | - | 437, 419, 164 |
| M52 | p, f, u | 467 | 399 | 354 | 326 | - | 337, 296, 251, 238, 221 |
| M53 | f | 437 | - | 338 | 310 | 268 | 352, 321, 283 |
| M54 | f | 437 | 369 | 338 | 310 | - | 321, 253, 251, 238, 205, 178 |
| M55 | p, f | 449 | 381 | 350 | 322 | - | 294, 217, 190 |
| M56 | f | 465 | 397 | 352 | 324 | - | 307, 219, 192 |
| M57 | f | 453 | 385 | 354 | 326 | - | 337, 251, 238, 221, 102 |
| M58 | p, f | 421 | 353 | 322 | 294 | - | 267, 162 |
| M59 | u | 627 | 397 | - | 310 | - | 465, 449, 378, 352, 336 |
| M60 | p, f | 433 | 365 | 320 | 292 | 250 | - |
| M61 | u | 543 | 299 | 254 | 226 | - | 367 |
| M62 | p, u | 643 | 381 | 338 | 310 | - | 437, 406, 378, 369 |
| M63 | p, f | 451 | 383 | 340 | 310 | - | 434, 423, 408, 338, 321, 312, 295, 294, 293, 281, 267 |
| M64 | p | 613 | 369 | 338 | - | - | 437 |
| M65 | p | 627 | 383 | 352 | - | - | 451, 336 |
| M66 | p | 435 | - | 322 | 294 | - | 189, 162 |
| ^a)^ Compilation of data from multiple LC-MS/MS analyses. | | | | | | | |
| Fragmentation of ribociclib | | | 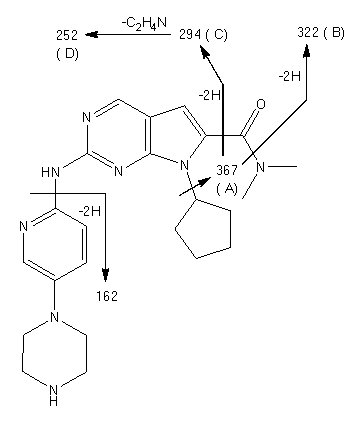 | | | | |

**Table S9. Summary of LC-MS exact mass and hydrogen/deuterium exchange data of [^14^C] ribociclib and metabolites observed in plasma, urine and feces**.

Data from LC-MS/MS runs of plasma, urine and feces from healthy male subjects following a single oral dose of 600 mg [^14^C] ribociclib. Metabolites listed in numerical order, proposed structures are shown in Table S10

| Compound ^a)^ | Biotransformation | Formula | [M+H]^+^ / [M-H]^-^ | Mass shift (mDa) | H/D exchange ^b)^ | Mass shift for H/D (mDa) |
| --- | --- | --- | --- | --- | --- | --- |
| Ribociclib | Parent | C_23_H_31_N_8_O | 435.2634 | 1.3 | +3 | 1.0 |
| M1 | C-hydroxylation and glucuronidation | C_29_H_39_N_8_O_8_ | 627.2906 | 1.5 | +7 | 0.9 |
| M4 (LEQ803) | N-demethylation | C_22_H_29_N_8_O | 421.2478 | 1.4 | +4 | 0.4 |
| M6 | N-oxygenation and glucuronidation | C_29_H_39_N_8_O_8_ | 627.2896 | 0.5 | +6 | 1.1 |
| M7 (CQM384) | N-demethylation and N-sulfation | C_22_H_29_N_8_O  C_22_H_29_N_8_O_4_S | 421.2469  501.2055 | 0.5  2.3 | +4 | 3.2 |
| M8 (CQM386) | N-sulfation | C_23_H_31_N_8_O_4_S | 515.2195 | 0.6 | +3 | 2.5 |
| M9 | Oxygenation – 2H | C_23_H_29_N_8_O_2_ | 449.2426 | 1.3 | +3 | 0.9 |
| M10 | C-hydroxylation | C_23_H_31_N_8_O_2_ | 451.2576 | 0.6 | +4 | 0.8 |
| M11 | N-demethylation and N-acetylation of piperazine | C_24_H_31_N_8_O_2_ | 463.2575 | 0.5 | +3 | 0.6 |
| M13 (CCI284) | N-hydroxylation | C_23_H_31_N_8_O_2_ | 451.2560 | 1.0 | ND | ND |
| M15 | N-methyl hydroxylation | C_23_H_31_N_8_O_2_ | 451.2581 | 1.1 | +4 | 0.2 |
| M18 | +CO | C_24_H_31_N_8_O_2_ | 463.2582 | 1.2 | +2 | 0.5 |
| M19 | N,N-dealkylation | C_21_H_29_N_8_O | 409.2469 | 0.5 | +5 | 0.7 |
| M20 | N-dealkylation of piperazine to aniline | C_19_H_24_N_7_O | 366.2053 | 0.8 | +4 | 1.0 |
| M21 | N-dealkylation of piperazine to aniline + N-acetylation | C_21_H_26_N_7_O_2_ | 408.2150 | 0.2 | +3 | 0.9 |
| M24 | N-dealkylation of piperazine to aniline, N-demethylation and N-acetylation | C_20_H_24_N_7_O_2_ | 394.2003 | 1.2 | +4 | 0.5 |
| M25 | N-demethylation + oxygenation | C_22_H_29_N_8_O_2_ | 437.2397 | 1.6 | ND | ND |
| M26 | 2 × C-hydroxylation and glucuronidation | C_29_H_39_N_8_O_9_ | 643.2845 | 0.5 | +8 | 1.4 |
| M27 | Dehydrogenation | C_23_H_29_N_8_O | 433.2467 | 0.3 | +2 | 1.1 |
| M28 | N-demethylation, Oxygenation – 2H | C_22_H_27_N_8_O_2_ | 435.2269 | 1.2 | +4 | 1.0 |
| M29 | N-demethylation + N-oxygenation | C_22_H_29_N_8_O_2_ | 437.2391 | 2.2 | +4 | 0.5 |
| M30 | N-demethylation and N-oxygenation | C_22_H_29_N_8_O_2_ | 437.2416 | 0.3 | +4 | 0.8 |
| M31 | N-demethylation and N,N-dealkylation | C_20_H_27_N_8_O | 395.2312 | 0.4 | +6 | 0.2 |
| M32 | N-demethylation, N-oxygenation and glucuronidation | C_28_H_37_N_8_O_8_ | 613.2750 | 1.6 | +7 | 0.2 |
| M33 | N-dealkylation of piperazine to aniline, N-acetylation and C-hydroxylation | C_21_H_26_N_7_O_3_ | 424.2106 | 0.9 | +4 | 0.7 |
| M34 | Cysteine conjugate | C_26_H_36_N_9_O_3_S | 554.2670 | 0.8 | +6 | 1.4 |
| M35 | Oxygenation and glucuronidation | C_29_H_39_N_8_O_8_ | 627.2870 | 2.1 | ND | ND |
| M37 | 2 × C-hydroxylation | C_23_H_31_N_8_O_3_ | 467.2510 | 0.9 | +5 | 0.5 |
| M38 | Cysteine conjugate | C_26_H_36_N_9_O_3_S | 554.2668 | 0.6 | +6 | 1.3 |
| M41 | C-hydroxylation | C_23_H_31_N_8_O_2_ | 451.2574 | 0.4 | +4 | 0.5 |
| M42 | N-dealkylation of piperazine and C-hydroxylation | C_21_H_28_N_7_O_2_ | 410.2317 | 1.3 | +4 | 0.8 |
| M43 | C-hydroxylation, N-oxygenation and glucuronidation | C_29_H_39_N_8_O_9_ | 643.2845 | 0.5 | +7 | 0.3 |
| M44 | N-dealkylation and C-hydroxylation | C_14_H_20_N_5_O_2_ | 290.1626 | 0.9 | +4 | 0.4 |
| M46 | N-demethylation, C-hydroxylation, Oxygenation – 2H | C_22_H_27_N_8_O_3_ | 451.2218 | 1.2 | +5 | 1.3 |
| M47 | C-hydroxylation, N-oxygenation, dehydrogenation and glucuronidation | C_29_H_37_N_8_O_9_ | 641.2695 | 1.2 | +6 | 1.8 |
| M48 | N-demethylation + cysteine conjugate | C_25_H_34_N_9_O_3_S | 540.2516 | 1.1 | +7 | 0.7 |
| M49 | N-demethylation + oxygenation and loss of CH_2_ from piperazine | C_21_H_27_N_8_O_2_ | 423.2267 | 1.0 | +5 | 0.3 |
| M50 | 2 × oxygenation – 4H | C_23_H_27_N_8_O_3_ | 463.2208 | 0.2 | +3 | 0.9 |
| M51 | 2 × C-hydroxylation, 1 × oxygenation – 2H | C_23_H_29_N_8_O_4_ | 481.2320 | 0.8 | +5 | 1.6 |
| M52 | 2 × C-hydroxylation | C_23_H_31_N_8_O_3_ | 467.2527 | 0.8 | +5 | 1.2 |
| M53 | N-demethylation and C-hydroxylation | C_22_H_29_N_8_O_2_ | 437.2423 | 1.0 | +5 | 0.8 |
| M54 | N-demethylation and C-hydroxylation | C_22_H_29_N_8_O_2_ | 437.2419 | 0.6 | +5 | 1.3 |
| M55 | N-demethylation + CO | C_23_H_29_N­_8_O_2_ | 449.2421 | 0.8 | +3 | 0.5 |
| M56 | + CH_2_O | C_24_H_33_N_8_O_2_ | 465.2725 | 0.1 | +4 | 0.0 |
| M57 | N-Demethylation and 2 × C-hydroxylation | C_22_H_29_N_8_O_3_ | 453.2366 | 0.3 | +6 | 0.4 |
| M58 | N-demethylation + O -2H – CH_2_ | C_21_H_25_N_8_O_2_ | 421.2111 | 1.1 | +4 | 1.3 |
| M59 | N-Oxygenation + C-oxygenation – 2H + glycosylation | C_29_H_39_N_8_O_8_ | 627.2886 | 0.5 | +6 | 0.5 |
| M60 | Dehydrogenation | C_23_H_29_N_8_O | 433.2470 | 0.6 | +2 | 0.6 |
| M61 | Loss of piperazine ring, C-hydroxylation and glucuronidation | C_25_H_31_N_6_O_8_ | 543.2219 | 1.6 | +6 | 1.0 |
| M62 | C-hydroxylation, N-oxygenation and glucuronidation | C_29_H_39_N_8_O_9_ | 643.2855 | 1.5 | +7 | 0.0 |
| M63 | C-hydroxylation | C_23_H_31_N_8_O_2_ | 451.2574 | 0.4 | +4 | 0.4 |
| M64 | N-demethylation, oxygenation and glucuronidation | C_28_H_37_N_8_O_8_ | 613.2712 | 2.2 | ND | ND |
| M65 | N-demethylation, 2 × oxygenation, dehydrogenation and glucuronidation | C_28_H_35_N_8_O_9_ | 627.2515 | 1.2 | ND | ND |
| M66 | Oxidation and decarboxylation of piperazine, + oxygenation – 4H | C_22_H_27_N_8_O_2_ | 435.2242 | 1.5 | ND | ND |
| ^a)^ Compilation of data from multiple LC-MS/MS analyses  ^b)^ Mass shift observed with Hydrogen / Deuterium exchange LC/MS | | | | | | |

**Table S10. Structures of ribociclib and its metabolites identified in human**

Only metabolites subsequently found in the human ADME study are listed

| Compound | Structure | Biotransformation | MW | Mass difference to ribociclib |
| --- | --- | --- | --- | --- |
| ribociclib |  | - | 434.3 | - |
| M1 |  | C-hydroxylation, glucuronidation | 626.3 | +192 |
| M4 (LEQ803) |  | N-demethylation | 420.3 | -14 |
| M6 |  | oxygenation, glucuronidation | 626.3 | +192 |
| M7  (CQM384) |  | N-demethylation, sulfation | 500.3 | +66 |
| M8  (CQM386) |  | sulfation | 514.3 | +80 |
| M9 |  | oxygenation, oxidation (-2H) | 448.3 | +14 |
| M10 |  | C-hydroxylation | 450.3 | +16 |
| M11 |  | N-demethylation, N-acetylation | 462.3 | +28 |
| M13  (CCI284) |  | N-hydroxylation | 450.3 | +16 |
| M15 |   Most likely structure | C-hydroxylation | 450.3 | +16 |
| M18 |  | N-formylation | 462.3 | +28 |
| M19 |  | N-N-dealkylation | 408.3 | -26 |
| M20 |  | N-dealkylation | 365.2 | -69 |
| M21 |  | -C_4_H_7_N + acetylation | 407.2 | -27 |
| M24 |  | -C_4_H_7_N + demethylation + acetylation | 393.2 | -41 |
| M25 |  | oxygenation + demethylation | 436.2 | +2 |
| M26 |  | 2 oxygenations + glucuronidation | 642.3 | +208 |
| M27 |  | desaturation | 432.3 | -2 |
| M28 |  | oxygenation + desaturation + demethylation | 434.2 | 0 |
| M29 |  | oxygenation + demethylation | 436.2 | +2 |
| M30 |  | oxygenation + demethylation | 436.2 | +2 |
| M31 |  | -C_2_H_2_ + demethylation | 394.2 | -40 |
| M32 |  | oxygenation + demethylation + glucuronidation | 612.3 | +178 |
| M33 |  | -C_4_H_7_N + oxygenation + acetylation | 423.2 | -11 |
| M34 |  | cysteine conjugation | 553.3 | +119 |
| M35 |  | C-hydroxylation, glucuronidation | 626.3 | +192 |
| M37 |  | 2 x C-hydroxylation | 466.3 | +32 |
| M38 |  | cysteine conjugation | 553.3 | +119 |
| M41 |  | C-hydroxylation | 450.3 | +16 |
| M42 |  | C-hydroxylation, piperazine ring cleavage. | 409.2 | -25 |
| M43 |  | C-hydroxylation, N-oxygenation and glucuronidation | 642.3 | +208 |
| M44 |  | N-dealkylation and C-hydroxylation | 289.2 | -145 |
| M46 |  | N-demethylation, C-hydroxylation, oxygenation – 2H | 450.2 | +16 |
| M47 |  | C-hydroxylation, N-oxygenation, dehydrogenation and glucuronidation | 640.3 | +206 |
| M48 |  | N-demethylation + cysteine conjugate | 539.3 | +105 |
| M49 |  | N-demethylation + oxygenation and loss of CH_2_ from piperazine | 422.2 | -12 |
| M50 |  | 2 × oxygenation – 4H | 462.2 | +28 |
| M51 |  | 2 × C-hydroxylation, 1 × oxygenation – 2H | 480.2 | +46 |
| M52 |  | 2 × C-hydroxylation | 466.3 | +32 |
| M53 |  | -C_4_H_7_N + demethylation + acetylation + Oxygenation | 436.2 | +2 |
| M54 |  | N-demethylation and C-hydroxylation | 436.2 | +2 |
| M55 |  | N-demethylation + CO | 448.2 | +14 |
| M56 |  | + CH_2_O | 464.3 | +30 |
| M57 |  | N-demethylation and 2 × C-hydroxylation | 452.2 | +18 |
| M58 |  | N-demethylation + O -2H – CH_2_ | 420.2 | -14 |
| M59 |  | N-oxygenation + C-oxygenation – 2H + glycosylation | 626.3 | +192 |
| M60 |  | dehydrogenation | 432.2 | -2 |
| M61 |  | loss of piperazine ring, C-hydroxylation and glucuronidation | 542.2 | +108 |
| M62 |  | C-hydroxylation, N-oxygenation and glucuronidation | 642.3 | +208 |
| M63 |  | C-hydroxylation | 450.3 | +16 |
| M64 |  | N-demethylation, oxygenation and glucuronidation | 612.3 | +178 |
| M65 |  | N-demethylation, 2 × oxygenation, dehydrogenation and glucuronidation | 626.3 | +192 |
| M66 |  | oxidation and decarboxylation of piperazine, + oxygenation – 4H | 434.2 | 0 |

**Table S11. Cumulative total radiolabeled components excreted in urine and feces.**

Cumulative excretion of radioactivity in urine and feces after a single oral dose of 600 mg [^14^C]LEE011 to healthy male volunteers. (Source data for Figure 3 in manuscript)

|  | Mean excretion (n=6 subjects; % of dose) | | | | | |
| --- | --- | --- | --- | --- | --- | --- |
| Time (h) | Urine | SD | Feces | SD | Total | SD |
| Cumulative excretion of radioactivity in urine and feces per collection time period (% of dose) | | | | | | |
| 0 | 0 | 0 | 0 | 0 | 0 | 0 |
| 6 | 4.85 | 1.63 | 0 | 0 | - | - |
| 12 | 9.15 | 2.31 | 0 | 0 | - | - |
| 24 | 12.9 | 3.50 | 4.25 | 6.53 | 17.2 | 6.70 |
| 48 | 17.0 | 4.54 | 30.5 | 11.1 | 47.5 | 7.65 |
| 72 | 19.0 | 4.94 | 44.9 | 7.94 | 63.9 | 6.57 |
| 96 | 20.2 | 5.12 | 55.7 | 6.49 | 75.9 | 3.68 |
| 120 | 20.9 | 5.21 | 58.8 | 4.86 | 79.7 | 3.12 |
| 144 | 21.4 | 5.31 | 62.4 | 4.61 | 83.8 | 2.83 |
| 168 | 21.8 | 5.29 | 64.9 | 4.55 | 86.7 | 2.24 |
| 192 | 22.1 | 5.35 | 65.7 | 4.20 | 87.8 | 2.01 |
| 216 | 22.2 | 5.39 | 66.4 | 4.41 | 88.6 | 1.87 |
| 240 | 22.3 | 5.39 | 66.8 | 4.59 | 89.1 | 1.74 |
| 264 | 22.5 | 5.41 | 67.2 | 4.49 | 89.6 | 1.58 |
| 288 | 22.5 | 5.42 | 67.7 | 4.42 | 90.3 | 1.57 |
| 312 | 22.6 | 5.43 | 68.1 | 4.49 | 90.7 | 1.41 |
| 336 | 22.6 | 5.43 | 68.3 | 4.62 | 90.9 | 1.32 |
| 360 | 22.6 | 5.41 | 68.5 | 4.55 | 91.1 | 1.26 |
| 384 | 22.6 | 5.39 | 68.6 | 4.61 | 91.3 | 1.14 |
| 408 | 22.6 | 5.39 | 68.7 | 4.67 | 91.4 | 1.09 |
| 432 | 22.6 | 5.39 | 68.9 | 4.68 | 91.6 | 1.05 |
| 456 | 22.6 | 5.39 | 69.0 | 4.68 | 91.6 | 1.02 |
| 480 | 22.6 | 5.39 | 69.0 | 4.74 | 91.7 | 1.01 |
| 504 | 22.6 | 5.39 | 69.1 | 4.72 | 91.7 | 1.01 |
| SD: Standard deviation | | | | | | |
